# Carboxylesterase 2a deletion provokes hepatic steatosis and insulin resistance in mice involving impaired diacylglycerol and lysophosphatidylcholine catabolism

**DOI:** 10.1016/j.molmet.2023.101725

**Published:** 2023-04-12

**Authors:** Gabriel Chalhoub, Alina Jamnik, Laura Pajed, Stephanie Kolleritsch, Victoria Hois, Antonia Bagaric, Dominik Prem, Anna Tilp, Dagmar Kolb, Heimo Wolinski, Ulrike Taschler, Thomas Züllig, Gerald N. Rechberger, Claudia Fuchs, Michael Trauner, Gabriele Schoiswohl, Guenter Haemmerle

**Affiliations:** 1 Institute of Molecular Biosciences, University of Graz, Graz, Austria; 2 Division of Endocrinology and Diabetology, Medical University of Graz, Austria; 3 Department of Pharmacology and Toxicology, University of Graz, Graz, Austria; 4 Core Facility Ultrastructure Analysis, Medical University of Graz, Graz, Austria; 5 Hans Popper Laboratory of Molecular Hepatology, Division of Gastroenterology and Hepatology, Department of Internal Medicine III, Medical University of Vienna, Vienna, Austria

**Keywords:** NAFLD, Obesity, Carboxylesterase 2, Lipid signaling, Insulin resistance, DAG accumulation, Lysophosphatidylcholine

## Abstract

**Objective:**

Hepatic triacylglycerol accumulation and insulin resistance are key features of NAFLD. However, NAFLD development and progression are rather triggered by the aberrant generation of lipid metabolites and signaling molecules including diacylglycerol (DAG) and lysophosphatidylcholine (lysoPC). Recent studies showed decreased expression of carboxylesterase 2 (CES2) in the liver of NASH patients and hepatic DAG accumulation was linked to low CES2 activity in obese individuals. The mouse genome encodes several *Ces2* genes with *Ces2a* showing highest expression in the liver. Herein we investigated the role of mouse Ces2a and human CES2 in lipid metabolism *in vivo* and *in vitro*.

**Methods:**

Lipid metabolism and insulin signaling were investigated in mice lacking Ces2a and in a human liver cell line upon pharmacological CES2 inhibition. Lipid hydrolytic activities were determined *in vivo* and from recombinant proteins.

**Results:**

Ces2a deficient mice (Ces2a-ko) are obese and feeding a high-fat diet (HFD) provokes severe hepatic steatosis and insulin resistance together with elevated inflammatory and fibrotic gene expression. Lipidomic analysis revealed a marked rise in DAG and lysoPC levels in the liver of Ces2a-ko mice fed HFD. Hepatic lipid accumulation in Ces2a deficiency is linked to lower DAG and lysoPC hydrolytic activities in liver microsomal preparations. Moreover, Ces2a deficiency significantly increases hepatic expression and activity of MGAT1, a PPAR gamma target gene, suggesting aberrant lipid signaling upon Ces2a deficiency. Mechanistically, we found that recombinant Ces2a and CES2 show significant hydrolytic activity towards lysoPC (and DAG) and pharmacological inhibition of CES2 in human HepG2 cells largely phenocopies the lipid metabolic changes present in Ces2a-ko mice including reduced lysoPC and DAG hydrolysis, DAG accumulation and impaired insulin signaling.

**Conclusions:**

Ces2a and CES2 are critical players in hepatic lipid signaling likely via the hydrolysis of DAG and lysoPC at the ER.

## Highlights

• Carboxylesterase 2a (Ces2a) deletion provokes fatty liver disease in mice.
• Ces2a-ko deletion results in hepatic insulin resistance, DAG and lysoPC accumulation.
• Recombinant Ces2a and human CES2 efficiently hydrolyze DAG and lysoPC.
• CES2 inhibition in a human liver cell line (HepG2) phenocopies Ces2a deficiency.
• CES2 is rate-limiting in microsomal DAG hydrolysis in HepG2 cells.

## Graphical abstract

**Figure:**
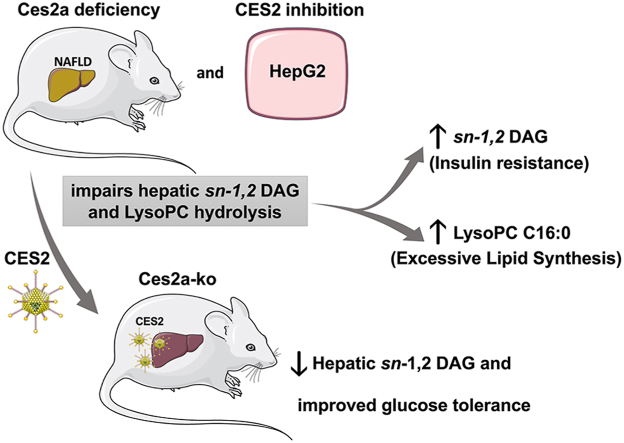

## 1 Introduction

Over the past decades, global obesity has nearly tripled and can nowadays be considered as a pandemic [1]. This drastic rise comes along with an increased incidence of obesity-related comorbidities such as type 2 diabetes, cardiovascular disease, and non-alcoholic fatty liver disease (NAFLD), making obesity the leading cause of premature death today. Already more than 25% of the world's population and over 90% of morbidly obese patients develop NAFLD [2]. NAFLD is defined as hepatic steatosis without liver injury, followed by aggressive non-alcoholic steatohepatitis (NASH) with liver fibrosis and ballooning [3,4].

Carboxylesterases 2 (Ces2/CES2) family members, which are well-known for their role in xenobiotic and (pro)drug metabolism [5,6], have been recently implicated in endogenous lipid and energy metabolism, especially in the context of obesity and metabolic stress [7,8]. CES2/Ces2 are α/β serine hydrolases primarily expressed in the liver and intestine [9]. They are soluble proteins mainly localized in the lumen of the ER due to their C-terminal ER retention sequence [10]. The human genome encodes one *CES2* gene as opposed to eight *Ces2* genes (*Ces2a-h* including one pseudogene) in mice [10]. In humans, low hepatic CES2 expression and activity have been demonstrated in NASH patients [7] and obese individuals [8], suggesting a role for CES2 in metabolic disease development. Likewise, low hepatic Ces2c expression has been linked to the development of fatty liver disease in mice as well [7], which has been reversed either by increasing hepatic Ces2c expression levels or ectopic expression of human CES2 [7]. However, among the Ces2 members, Ces2a shows the highest expression in the liver and is significantly downregulated upon high-fat diet (HFD)-induced metabolic stress [11], suggesting that Ces2a plays a critical role in liver lipid metabolism as well. To investigate the role of Ces2a in systemic energy homeostasis and hepatic lipid metabolism *in vivo*, we characterized global Ces2a knockout (Ces2a-ko) mice in the setting of diet-induced obesity. Ces2a deficiency provoked massive lipid accumulation in the liver, which was particularly prominent for *sn*-1,2 (2,3) diacylglycerols (DAGs) and lysoPC accompanied by impaired hepatic insulin signaling and glucose intolerance. Notably, inhibition of CES2 activity in a human liver cell line (HepG2) caused similar changes in lipid metabolism and insulin signaling as observed in the liver of Ces2a-ko mice, suggesting a related function of Ces2a and CES2 in hepatocytes.

## 2 Materials and methods

### 2.1 Animal models

Ces2a-ko mice were generated by transfection of mouse embryonic stem (ES) cells with a gene trap vector where exon 1 was partly deleted and resulted in an in-frame fusion with the *beta galactosidase* (*lacZ*) gene (Mutant Mouse Resource & Research Center, California, USA). These ES cells were injected into blastocysts and transferred into the uteri of C57BL6/N mice and founders were used to propagate the Ces2a-ko mouse strain on the full C57BL6/N background. Ces2a-ko and control mice were housed under a 14 h light - 10 h dark cycle in a specific pathogen-free environment with *ad libitum* access to food and water. Animals were fed a standard laboratory chow diet (11 kJ% fat; V1126, Ssniff, Germany) or HFD (45 kJ% fat; D12451, Ssniff, Germany) starting at 4–6 weeks of age. For genotyping, the following primers were used: *Ces2a-geno-fw*: 5’ CTGACTGTGTACCTATTGAC 3’, *Ces2a-geno-rv*: 5’ CATCAGCTGTCACCAC 3’, *LacZ-geno-rv*: 5’ GACGACGACAGTATCG 3’ generating the following PCR products: Ces2a-ko *LacZ*: 609 bp and WT *Ces2a*: 1030 bp. For adenoviral overexpression of CES2 or GFP, mice were fed HFD for 20 weeks and then injected with 1.6 × 10^9^ PFU particles of adenoviral vectors (Vector Biolabs) into the tail vein [8]. Six days post-injection, a glucose tolerance test (GTT) was performed. Eight days after injection, mice were sacrificed in *ad libitum* fed state. All animal experiments were approved by the Austrian Federal Ministry for Science, Research and Economy (protocol number BMBWF 2021–0.746.723) and conducted in compliance with the Council of Europe Convention (ETS 123). For all studies, male age-matched littermates were used. Genotype, age, feeding status, diet, and animal number are indicated for each experiment in the appropriate figure legends.

### 2.2 Metabolic and biochemical analyses

Mice were weighed weekly and body composition was determined every 4 weeks by nuclear magnetic resonance using TD-NMR miniSpec Live Mice Analyzer (Bruker Optics, Billerica, USA). Food intake, energy expenditure, respiratory exchange ratio, and locomotor activity were monitored using a laboratory animal monitoring system (Phenomaster, TSE Systems, Bad Homburg, Germany). Before metabolic phenotyping, mice were familiarized to drinking flasks and single housing for at least 24 h. Data were separated on the basis of light:dark cycle (average of 3–4 light:dark cycles).

For GTT, mice were injected intraperitoneally (ip) with 1.6 g glucose per kg body weight following a 6 h fast. Blood glucose before (0 min) and 15, 30, 60, and 120 min after injection was determined using a Wellion Calla glucometer (MedTrust, Marz, Austria). Blood ketone bodies were measured using Freestyle Optium KETONE Test Strips (Abbott Laboratories, Abbott Park, USA). Plasma lipid parameters were determined using the following commercial kits: fatty acids: HR Series NEFA-HR Reagents (Wako Diagnostics, USA), glycerol: Free glycerol reagent (Sigma–Aldrich, USA), triacylglycerol (TAG): Infinity Triglycerides Liquid Stable Reagent (ThermoFisher Scientific, USA), and total cholesterol (TC): DiaSys Diagnostic Systems (Holzheim, Germany). Plasma insulin was determined using Ultra-Sensitive Mouse Insulin ELISA Kit (Crystal Chem, USA) and plasma alanine aminotransferase (ALT) and aspartate aminotransferase (AST) levels were determined using Infinity reagents (ThermoFisher Scientific, USA). For analysis of VLDL secretion, HFD-fed mice were fasted overnight and then ip injected with 1 g Poloxamer 407 (Umforana, Wiesbaden, Germany) per kg of body weight. Blood samples were collected before (0) and 1, 2, 3, and 4 h after injection. Plasma TAG levels were determined using Infinity Triglycerides Liquid Stable Reagent (ThermoFisher Scientific).

### 2.3 X-Gal staining of mouse tissues

X-Gal staining allows the assessment of tissue-specific Ces2a expression since a LacZ reporter gene is included into the *Ces2a* gene. The staining was performed as described [12]. Tissues were fixed in 3.7% cold paraformaldehyde for 1 h at 4 °C. Afterwards, tissues were washed with rinse buffer (0.1 M Na_2_HPO_4_, 5 mM NaH_2_PO_4_, 3 mM MgCl_2_·6H_2_O, 1.5 mM sodium deoxycholate, 3% octylphenoxypolyethoxyethanol (IGEPAL CA-630) in phosphate-buffered saline; pH 7.5) and incubated in X-Gal staining solution (5 mM K_3_Fe(CN)_6_, 5 mM K_4_[Fe(CN)_6_]·3H_2_O, 1 mg/ml X-Gal solution in rinse buffer) overnight. After staining, tissues were fixed with 10% formalin for 1 h at room temperature and washed with 70% ethanol until bleaching.

### 2.4 Protein expression analysis

For protein expression, homogenized tissues were sonicated in ice-cold buffer A (0.25 M sucrose, 1 mM EDTA, 1 mM dithiothreitol, 20 μg/ml leupeptin, 2 μg/ml antipain and 1 μg/ml pepstatin; pH 7.4) supplemented with phosphatase inhibitor PhosStop [Roche]. Then, lysates were centrifuged at 1,000×*g* at 4 °C for 15 min. The supernatant was collected, and protein concentrations were determined by Bio-Rad Protein Assay (Bio-Rad, USA) using BSA as standard. Lysate proteins were denatured in SDS-loading dye at 95 °C, separated by SDS-PAGE (10% Tris-glycine), and transferred onto a PVDF membrane (pore size: 0.45 μm; Carl Roth, Karlsruhe, Germany) according to standard protocols. Membranes were blocked with 10% nonfat dry milk (Carl Roth) in 1xTST (50 mM Tris HCl, 0.1% Tween 20, 150 mM NaCl; pH 7.4). Primary antibodies used for protein expression are listed in Supplementary Table 1. Immunoblots were visualized using the Clarity Western ECL plus Western Blotting Detection Reagent (Fisher Scientific) and FUSION FX (VILBER Smart Imaging). Signal intensities were quantified by densitometric analyses using FUSION.Ink software.

### 2.5 Gene expression

Total RNA was extracted using TRIzol® reagent (Thermo Fisher Scientific), digested with DNaseI (New England Biolabs), and reverse-transcribed into cDNA using the LunaScript™ RT SuperMix Kit (New England Biolabs). Quantitative PCR (qPCR) was performed using the Universal SYBR Green Supermix (Bio-Rad) and the StepOnePlus™ system (Thermo Fisher Scientific). Relative mRNA levels were quantified using the ΔΔCT method with 36B4 as the reference gene. Primer sequences are listed in Supplementary Table 2.

### 2.6 Determination of hepatic lipids

Neutral lipids were extracted according to Folch [13]. Briefly, 20 mg of liver tissues were homogenized in 1 ml chloroform/methanol (2/1, v/v) using ball mill (Retsch GmbH, Germany). For phase separation, 200 μl H_2_O were added, samples were then thoroughly vortexed and centrifuged at 5,000×*g* at 4 °C for 10 min. The lower organic phase was collected and extraction was repeated using 500 μl of chloroform. The organic phases were combined and dried under N_2_ stream. Lipids were redissolved in 0.1% Triton X-100 using a thermomixer (Eppendorf, Germany) at 500 rpm at 37 °C overnight followed by sonication in a water bath sonicator (Transsonic T460, Elma). Acylglycerol and TC amounts were determined using the commercial kits Infinity Triglycerides Liquid Stable Reagent (ThermoFisher Scientific, USA) and DiaSys Diagnostic Systems (Holzheim, Germany), respectively. Infranatants were dried and dissolved in 0.1% SDS/0.3 N NaOH for 4 h to determine protein concentration using Pierce™ BCA protein assay kit (Thermo Fisher Scientific Inc.) using BSA as standard. To analyze neutral lipids by TLC, extracted lipids were dissolved in chloroform and separated by a two solvent system (First solvent: chloroform/acetone/glacial acetic acid [88/12/1, v/v/v] was used for lipid separation for two-thirds of a silica plate. Second solvent: 100% toluene was used to separate lipids on the whole silica plate). The dried plate was then dipped in charring solution (25% ethanol, 10% H_3_PO_4_, 5% CuSO_4_) and incubated for 20 min at 140 °C followed by densitometric analysis.

For targeted lipidomic analysis, 20 mg liver tissue were extracted by 700 μl of MTBE supplemented with an internal standard. Therefore, samples were homogenized using a ball mill at 4 °C for 20 min. Then, 400 μl H_2_O were added and samples were vortexed immediately and mixed using a thermomixer at 4 °C for 15 min. Samples were then centrifuged at 4,000 rpm and 4 °C for 15 min. Hundred μl supernatant were collected, evaporated, and dissolved in 300 μl isopropyl alcohol/methanol/water (30/15/5, v/v/v). Chromatographic separation was performed using a 1290-UHPLC system (Agilent, Waldbronn, GER) equipped with a BEH-C18-column, 2.1 × 150 mm, 1.7 μm (Waters, Manchester, UK). A binary gradient was applied with water as solvent A and isopropyl alcohol as solvent B. Both solvents contained 8 μM phosphoric acid, 10 mM ammonium acetate, and 0.1 vol% formic acid. The linear gradient started at 50% solvent B at a constant flow rate of 0.15 ml/min and increased to 100% solvent B within 20 min. In the following 4 min solvent B percentage was kept at 100%. The column was re-equilibrated for 5 min, resulting in a total HPLC run time of 30 min. A 4670 triple quadrupole mass spectrometer (Agilent) equipped with an ESI source was used for analysis. Samples were analyzed in MRM mode and data analysis was done using the MassHunter 10.0 software package (Agilent). Protein concentration was determined by Pierce™ BCA protein assay kit (Thermo Fisher Scientific Inc.) with BSA as standard.

### 2.7 Isolation of microsomal fraction

Twenty mg of liver tissues were homogenized in ice-cold buffer A and subsequently centrifuged at 1,000×*g* and 4 °C for 10 min. The supernatants were collected and overlaid with ice-cold overlay buffer (100 mM potassium phosphate buffer; pH 7.4) and centrifuged at 100,000×*g* and 4 °C for 1 h using optima L 90-K ultracentrifuge (Beckman, California, USA). Microsomal fraction (pellet) was dissolved in 100–200 μl of ice-cold buffer A supplemented with 0.1% Triton X-100 and sonicated with an amplitude of 15% for 2 × 10 s. Protein concentrations were determined by Bio-Rad Protein Assay (Bio-Rad, USA) using BSA as standard.

### 2.8 Activity assays

TAG and diacylglycerol (DAG) hydrolase activity assays were performed in the presence of 25 mM CHAPS as described previously [11] with some modifications. For TAG hydrolase assay, 25 μg of microsomal liver fractions were incubated with 1.67 mM triolein (TAG C18:1; T7140 Sigma–Aldrich) and radiolabeled triolein as tracer [9,10-^3^H] (PerkinElmer Vertriebs GmbH, Traiskirchen, Austria). DAG hydrolase activity assay was performed by incubating 25 μg of microsomal liver fractions [11] in buffer A either with 2 mM of *sn*-1,2 (DAG C18:1; D0138, Sigma–Aldrich) or *sn*-1,3 dioleoyl-glycerol (DAG C18:1; D3627, Sigma–Aldrich). Hydrolase activities were determined by measuring FA release using HR Series NEFA-HR Reagents (Wako Diagnostics, USA). For lysophosphatidylcholine (lysoPC) hydrolase activity assay, 25 μg of microsomal fraction in 50 μl buffer A were incubated with 50 μl of 2 mM lysoPC (lysoPC 16:0; 855675, Avanti Polar Lipids) for 1 h at 37 °C. The substrate was prepared by sonication in assay buffer (100 mM potassium phosphate buffer, 50 mM KCl; pH 7.4) supplemented with 5 mM CHAPS as a detergent. After sonication, FA-free BSA (Sigma–Aldrich) was added to get a final concentration of 1%. The assay was stopped at 75 °C for 10 min, and FA release was measured to determine lysoPC hydrolase activity. MGAT and DGAT activity assays were performed as described previously [14] with some modifications. Fifty μl of microsomal fraction containing 20 μg protein were incubated with 100 μl of ice-cold acyltransferase assay buffer (5 mM MgCl_2_, 1.25 mg/ml BSA, 200 mM sucrose, 100 mM Tris⋅HCl; pH 7.4) containing either 0.2 mM *sn-*2 oleoyl glycerol (MAG C18:1; M2787, Sigma–Aldrich) to measure MGAT activity or 0.2 mM *sn*-1,2 dioleoyl-glycerol (DAG C18:1; D0138, Sigma–Aldrich) to measure DGAT activity in the presence of oleoyl [1–^14^C] coenzyme A (ARC0527, American Radiolabeled Chemicals) for 20 min at 25 °C. The reaction was stopped by adding 1 ml chloroform/methanol (2/1, v/v) and lipids were separated by TLC using hexane/diethyl ether/glacial acetic acid (70/29/1, v/v/v) as solvent. DAG and TAG bands were stained with iodine and cut to measure the radioactivity by liquid scintillation counting.

For Ces2a/CES2 protein hydrolytic activity assays, 5 μg of the purified proteins in 50 μl buffer A were incubated with 50 μl of either 2 mM PA (PA 18:1; 840875, Avanti Polar Lipids), PC (PC 18:1; 850375, Avanti Polar Lipids), PE (PE 18:1; 850725, Avanti Polar Lipids), PG (PG 18:1; 840475, Avanti Polar Lipids), PI (Liver PI; 840042, Avanti Polar Lipids), PS (PS 18:1; 840035, Avanti Polar Lipids), lysoPC (lysoPC 16:0: 855675, Avanti Polar Lipids; lysoPC 18:0: 855775, Avanti Polar Lipid; lysoPC 18:1: 845875, Avanti Polar Lipids) for 1 h at 37 °C. The substrates were prepared by sonication in assay buffer (100 mM potassium phosphate buffer, 50 mM KCl; pH 7.4) supplemented with 5 mM CHAPS and FA-free BSA (Sigma–Aldrich) was added to get a final concentration of 1%. The assays were stopped at 75 °C for 10 min and FA release was measured. In addition, lysoPC 16:0 hydrolase activity of purified CES2 was determined in the presence of 1 μl Loperamide as specific CES2 inhibitor and DMSO as control. DAG hydrolase activity assay of purified CES2 was performed by incubating 5 μg of CES2 in 50 μl buffer A with 2 mM of 1,2-1,3 dioleoyl-glycerol mixture (DAG C18:1; D8894, Sigma–Aldrich) in the presence of 1 μM Loperamide or DMSO for 1 h at 37 °C and the assay was stopped at 75 °C for 10 min. DAG substrate was prepared by sonication in assay buffer supplemented with 25 mM CHAPS and 1% FA-free BSA. The DAG hydrolase activity of purified CES2 was determined by measuring the FA release.

### 2.9 Liver histology

Liver was fixed in 4% neutral-buffered formaldehyde solution for 24 h and embedded in paraffin (Tissue Tek Tec, Sakura). Samples were sectioned and stained with hematoxylin and eosin (H&E) using standard histological protocols.

### 2.10 Cell culture

HepG2 cells (ECACC, #85011430) were cultured in DMEM low glucose media (1 g/l; Gibco) supplemented with 10% FBS and 1% Pen/Strep. To inhibit CES2 activity, cells were incubated with 10 μM Loperamide (Sigma–Aldrich) or DMSO as control for 48 h. For insulin signaling, cells were incubated with depleting media (DMEM low glucose) for 2 h followed by a 30 min treatment with 10 μg/ml insulin in depleting media. Protein and gene expression analyses were performed as described above. For TLC, neutral lipids were extracted from cells using hexane/isopropyl alcohol (3/2, v/v). To improve purity, lipids were re-extracted according to Folch using 2 × 1 ml chloroform/methanol (2/1, v/v) and then separated by TLC as described above. For DAG hydrolase activity assay, 25 μg of microsomal fraction were incubated with 2 mM 1,2-1,3 dioleoyl-glycerol (DAG C18:1; D8894, Sigma–Aldrich) and radiolabeled 1,3 dioleoyl-glycerol [1,2,3-^3^H] (DAG C18:1; ARC1604, American Radiolabeled Chemicals) as tracer in the presence of 2 μM Loperamide or DMSO as control for 1 h at 37 °C. LysoPC hydrolase activity assay was performed as described above with some modifications to avoid the turbidity of Loperamide that may affect the photometric detection. Fifty μg of HepG2 microsomal fraction were incubated in the presence of 2 μM Loperamide or DMSO as a control. The assay was stopped by adding extraction solution I (methanol/chloroform/n-heptane [10/9/7, v/v/v]) and extraction solution II (0.1 M potassium carbonate, pH 10.5) to separate phases, then 50 μl from the upper phase containing FAs were used to measure the lysoPC hydrolytic activity using commercial NEFAs assay kit. For lipid droplet staining, HepG2 cells were seeded in 35 mm glass bottom dishes (Ibidi), incubated with Loperamide or DMSO for 48 h. Cellular lipid droplets were stained with 0.38 μM BODIPY 493/503 (ThermoFisher Scientific) for 25 min. Nuclei of the cells were stained with 0.38 μM DAPI (Roche) for 20 min. Microscopy was performed using a Leica SP8 confocal microscope (Leica Microsystems Inc.) with spectral detection and a Leica HC FLUOTAR 25x/NA 0.95 W VISIR objective. BODIPY 493/503 was exited at 488 nm and detected between 500 and 550 nm. DAPI was excited at 405 nm and detected between 410 and 475 nm. At least 20 positions at the sample slide were selected arbitrarily and z-stacks acquired using 80 × 80 × 250 nm sampling. Deconvolution of the 3D data was performed using Huygens Pro (SVI imaging, The Netherlands), a theoretical point-spread-function and a low number of three iterations. Further image processing was carried out using the open-source software Fiji [15]. Background of 3D deconvolution data was subtracted (subtract background feature; rolling ball radius: 5 pixels). Subsequently, the data was projected using the maximum-intensity projection method. LDs were binarized using the Otsu method. A watershed algorithm was applied to separate closely associated image objects. The total area of segmented LDs was computed. LDs were counted by detection of the central maxima of the diffraction pattern of spherical LDs according to [16]. Finally, the mean LD area (μm^2^) was determined as total LD area per total LD counts. All cell culture studies were performed 3 times with a minimum of 3 biological replicates for each study.

### 2.11 Statistical analysis

Data are expressed as means ± SEM. Statistical significance was determined by unpaired Student's two-tailed *t* test or one-way or two-way ANOVA followed by Tukey's or Bonferroni's post hoc analysis. For lipidomic analysis, data (normalized to an internal standard and to protein content) were log2-transformed before using Shapiro–Wilk [17] to test normal distribution. As not all data showed normal distribution, we used the nonparametric Wilcoxon rank sum test in combination with the Benjamin-Hochburg [18] procedure for multiple test correction. Differences between groups were considered statistically significant for ∗ P < 0.05, ∗∗P < 0.01, and ∗∗∗P < 0.001.

## 3 Results

### 3.1 Ces2a deficiency provokes adiposity

**Figure 1:**
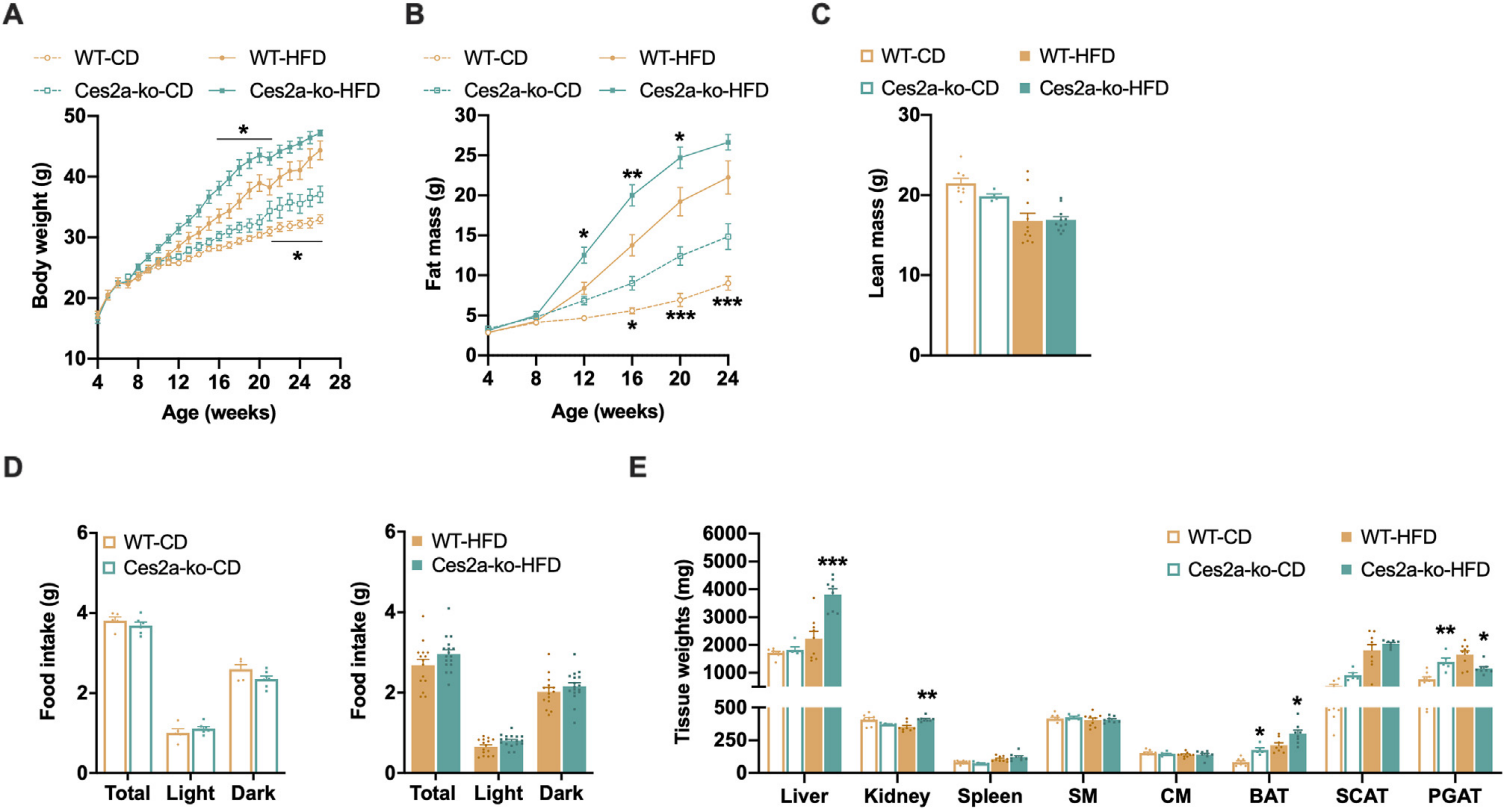
**Impact of Ces2a deficiency on systemic energy homeostasis.** (**A**) Longitudinal body weight and (**B**) fat mass in wild-type (WT) and Ces2a knockout (Ces2a-ko) mice (M, 4–24 weeks, chow, HFD, *ad libitum* fed, n = 7–12). (**C**) Lean mass (M, 26–28 weeks, chow, HFD, *ad libitum* fed, n = 5–11). (**D**) Food intake of single-housed mice per day fed chow (left) or HFD (right; M, 15 weeks, *ad libitum* fed, n = 5–17). (**E**) Tissue weights (SM, skeletal muscle [quadriceps]; CM, cardiac muscle, adipose tissue: BAT, brown; SCAT, subcutaneous; PGAT, perigonadal. M, 26–28 weeks, chow, HFD, *ad libitum* fed, n = 5–9). Data represent mean ± SEM. Statistical significance was determined by A, B, D) two-way ANOVA followed by Bonferroni's *post hoc* analysis or C, E) one-way ANOVA followed by Tukey's *post hoc* analysis. P values compare effect of genotype: ∗P < 0.05, ∗∗P < 0.01, ∗∗∗P < 0.001. (For interpretation of the references to color in this figure legend, the reader is referred to the Web version of this article.)

We have recently demonstrated that Ces2a, the most abundantly expressed *Ces2* gene in the mouse liver, is a potent TAG, DAG, and monoacylglycerol (MAG) hydrolase [11]. To investigate the role of Ces2a on systemic and liver lipid metabolism *in vivo*, we generated mice globally lacking Ces2a (Ces2a-ko mice) by inserting the *lacZ* gene into exon one of the *Ces2a* gene leading to non-detectable *Ces2a* mRNA expression (Fig. S1A). X-Gal staining of multiple tissues obtained from Ces2a-ko mice corroborate that Ces2a is primarily expressed in the liver and the intestine but not in other tissues such as adipose tissue (AT; Fig. S1B). Ces2a deletion provoked increased body weight and fat mass gain when fed chow or HFD (Figure 1A, B), whereas lean mass was comparable to WT (Figure 1C). Despite changes in body weight and body composition, metabolic phenotyping of Ces2a-ko mice revealed no differences in total energy expenditure, respiratory exchange ratio, locomotor activity, and food intake (Figure S1C–E and Figure 1D). Plasma fatty acid, glycerol, and ketone body levels were comparable between the genotypes irrespective of diet and feeding status. In contrast, plasma cholesterol levels were increased in Ces2a-ko mice fed chow or HFD, while plasma TAG levels were reduced in fasted and refed HFD-fed Ces2a-ko mice (Table 1 and Table S3). Although Ces2a was not detectable in AT, Ces2a-ko mice exhibited increased brown AT mass irrespective of diet and showed differences in regional fat distribution of white AT depots including increased perigonadal AT mass on chow diet but decreased perigonadal AT weight on HFD (Figure 1E). Notably, liver weight was markedly (1.7-fold) increased in Ces2a-ko mice when fed HFD (Figure 1E). Together, Ces2a deficiency provokes increased adiposity in mice independent of the diet and despite normal food intake and energy expenditure.

**Table 1:** Plasma parameters of WT and Ces2a-ko mice fed HFD.

	WT-HFD	Ces2a-ko-HFD	WT-HFD	Ces2a-ko-HFD
Parameter	**fasted**		**refed**	
Fatty acid (mM)	1.08 ± 0.05	1.04 ± 0.05	1.11 ± 0.10	1.29 ± 0.08
Glycerol (mM)	0.52 ± 0.03	0.45 ± 0.04	0.68 ± 0.06	0.66 ± 0.03
Triacylglycerol (mM)	0.99 ± 0.05	0.77 ± 0.02∗∗∗	2.26 ± 0.08	1.91 ± 0.10∗
Total cholesterol (mM)	4.01 ± 0.33	5.46 ± 0.16∗∗	3.98 ± 0.31	5.52 ± 0.17∗∗∗
Ketone bodies (mM)	1.71 ± 0.15	1.95 ± 0.14		

Plasma parameters of WT and Ces2a-ko mice fed HFD (M, 26 weeks, fasted 12 h, 2 h refed n = 8). Data represent mean ± SEM. Statistical significance was determined by Student's two-tailed t test. P values compare effect of genotype: P < 0.05: ∗, P < 0.01: ∗∗, P < 0.001: ∗∗∗.

### 3.2 Ces2a deficiency aggravates diet-induced hepatic steatosis with features of liver fibrosis and injury

**Figure 2:**
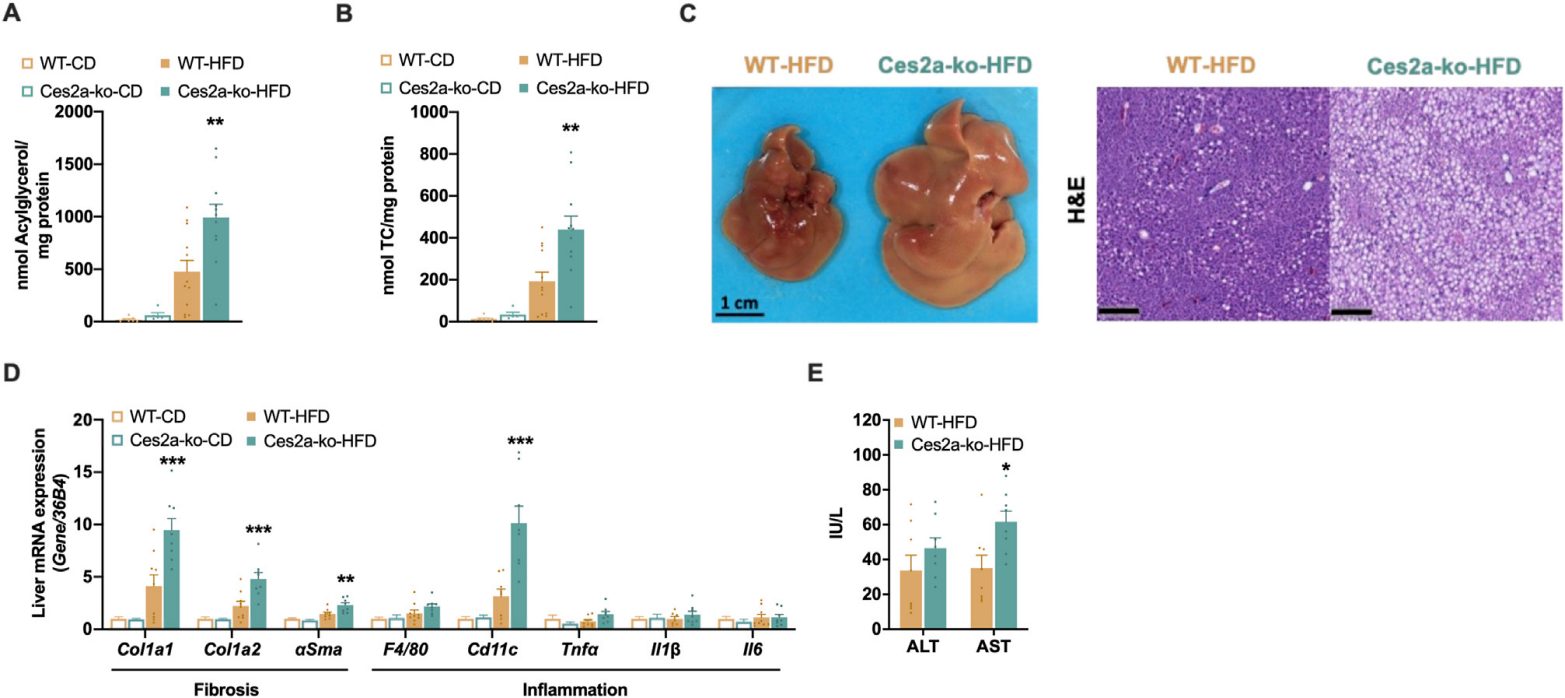
**Impact of Ces2a deficiency on liver lipid metabolism, inflammation, and injury.** (**A**) Hepatic acylglycerol and (**B**) total cholesterol (TC) content (M, 26–28 weeks, chow, HFD, *ad libitum* fed, n = 5–12). (**C**) Representative gross images (scale bar: 1 cm) and histological staining of liver sections with H&E (scale bar: 200 μm; M, 26 weeks, HFD, *ad libitum* fed). (**D**) Hepatic mRNA expression of fibrosis and immune cell markers and genes involved in inflammation relative to *36B4* reference gene by qPCR with WT mice arbitrarily set to 1 for each gene (M, 26–28 weeks, chow, HFD, *ad libitum* fed, n = 5–9). (**E**) Plasma ALT and AST levels (M, 26 weeks, HFD, *ad libitum* fed, n = 8). Data represent mean + SEM. Statistical significance was determined by A, B, D) one-way ANOVA followed by Tukey's *post hoc* analysis or E) Student's two-tailed *t* test. P values compare effect of genotype: ∗P < 0.05, ∗∗P < 0.01, ∗∗∗P < 0.001.

Consistent with increased liver weight upon HFD feeding, hepatic acylglycerol and total cholesterol content were significantly increased in HFD-fed Ces2a-ko mice compared to WT mice but unchanged in chow diet fed mice (Figure 2A, B). Gross images and microscopic staining corroborated lipid accumulation in the liver of HFD-fed Ces2a-ko mice by its yellow color and the presence of enlarged hepatic lipid droplets (Figure 2C). Augmented lipid deposition in Ces2a-ko mice on HFD was accompanied by increased hepatic mRNA expression of genes involved in fibrosis (*Col1a1, Col1a2, αSMA*) and inflammation including the immune cell marker *Cd11c* (Figure 2D). Likewise, plasma ALT and AST levels tended or were significantly higher in HFD-fed Ces2a-ko mice (Figure 2E).

**Figure 3:**
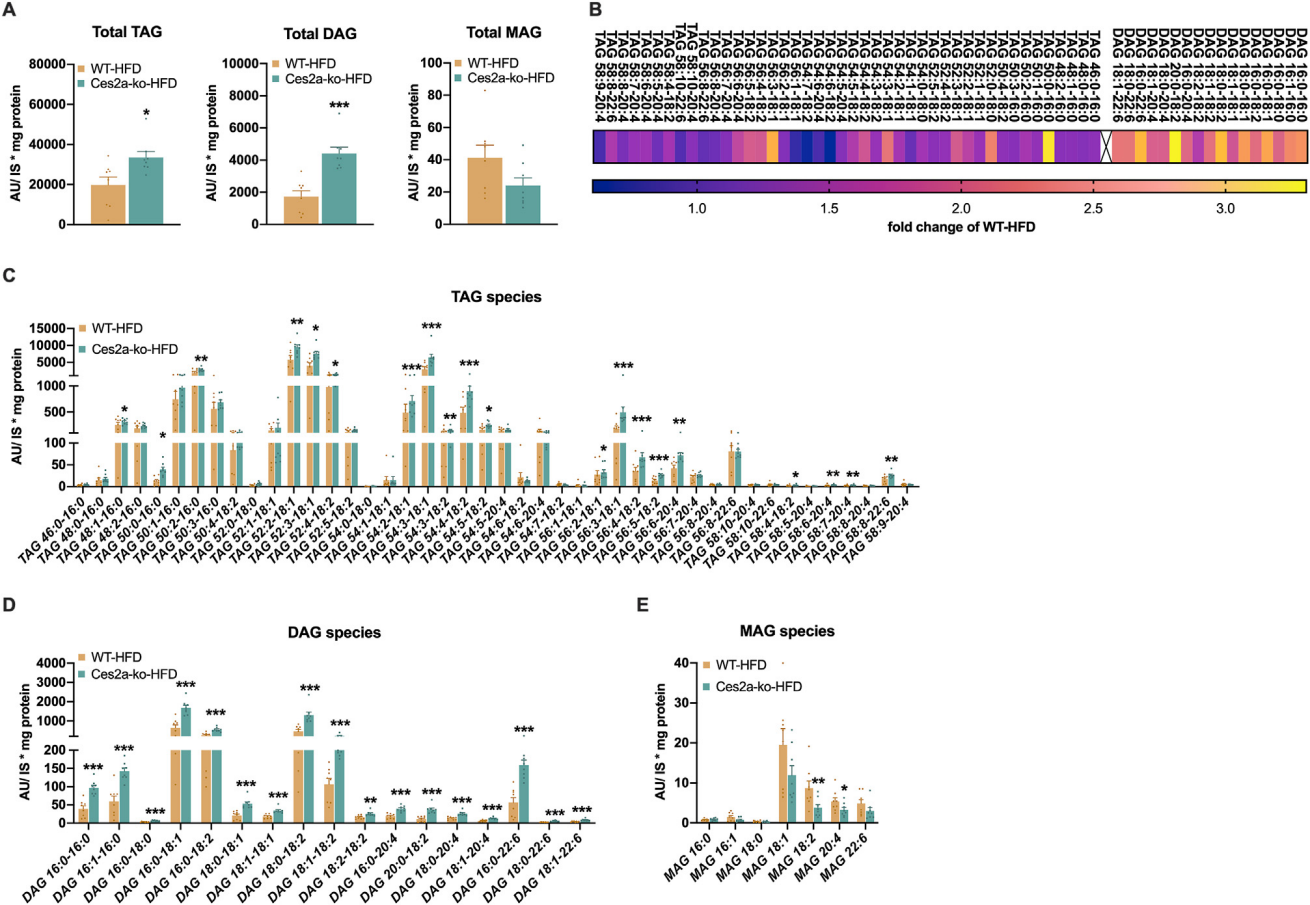
**Impact of Ces2a deficiency on liver lipid species.** Lipidomic analysis of the liver of HFD-fed mice (M, 26 weeks, *ad libitum* fed, n = 6). (**A**) Total hepatic triacylglycerol (TAG, left), diacylglycerol (DAG, middle), and monoacylglycerol (MAG, right) levels. (**B**) Heatmap of hepatic TAG and DAG lipid species as fold change of WT-HFD. Hepatic (**C**) TAG, (**D**) DAG, and (**E**) MAG species (M, 26 weeks, HFD, *ad libitum* fed, n = 6). Data represent mean + SEM. Statistical significance was determined by A) Student's two-tailed *t* test or C, D, E) non-parametric Wilcoxon rank sum test in combination with the Benjamin-Hochburg procedure for multiple test correction. P values compare effect of genotype: ∗P < 0.05, ∗∗P < 0.01, ∗∗∗P < 0.001.

A detailed lipidomic analysis of the liver of HFD-fed mice revealed a significant increase in total TAG (1.7 fold) and DAG levels (2.6 fold) in Ces2a-ko mice, whereas MAG levels tended to be reduced in the liver (Figure 3A). Notably, all measured DAG species - irrespective of saturation level and length - were increased in the liver of Ces2a-ko mice fed HFD (Figure 3B, D), whereas only a couple of hepatic TAG species were elevated (Figure 3B, C), and their increase was not as pronounced as compared to DAG species. In contrast, MAG species were reduced albeit not all species reached statistical significance (Figure 3E). Furthermore, total ceramide levels tended to be increased in the liver of HFD-fed Ces2a-ko mice leading to a significant upregulation of some ceramide species (Fig. S2A). Thus, deletion of Ces2a exacerbates the development of hepatic steatosis including massive DAG accumulation upon diet-induced obesity.

### 3.3 Ces2a deficiency impairs *sn*-1,2 DAG hydrolysis and activates lipid synthesis

**Figure 4:**
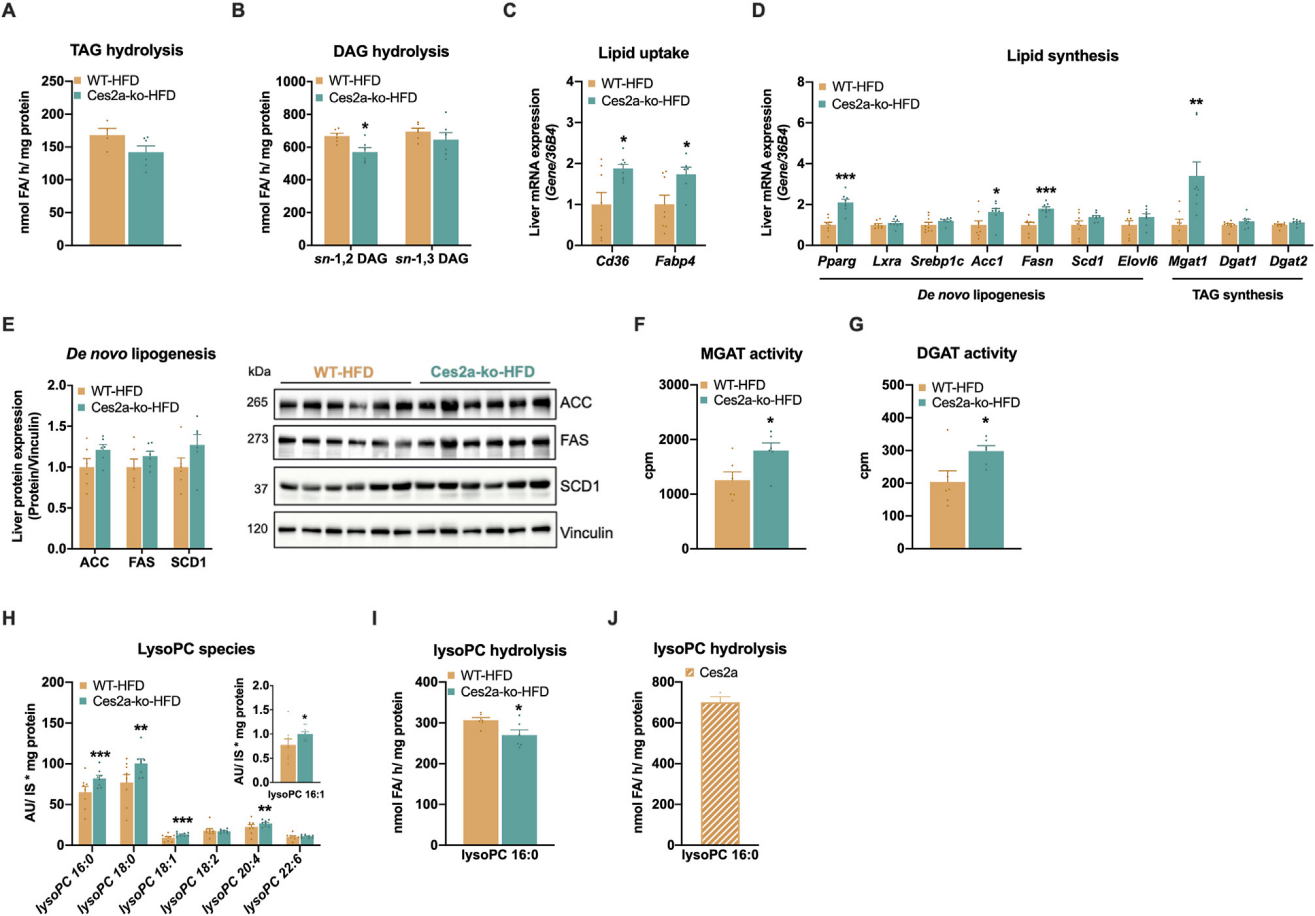
**Ces2a deficiency interferes with** hepatic lipid metabolism. **(A–B)** Neutral lipid hydrolase activity assays using microsomal liver fractions. (**A**) TAG hydrolase activity assay and (**B**) DAG hydrolase activity assay using *sn*-1,2 or *sn*-1,3 dioleoyl-glycerol (M, 26 weeks, HFD, *ad libitum* fed, n = 6). Hepatic mRNA expression of genes involved in (**C**) lipid uptake and (**D**) lipid synthesis relative to *36B4* reference gene by qPCR with WT mice arbitrarily set to 1 for each gene (M, 26 weeks, HFD, *ad libitum* fed, n = 8). (**E**) Hepatic expression of proteins involved in *de novo* lipogenesis. Left: Quantification of ACC, FAS, and SCD1 relative to Vinculin. Right: Representative immunoblots (M, 26 weeks, HFD, *ad libitum* fed, n = 6). (**F**) MGAT and (**G**) DGAT activity assay using microsomal liver fractions (M, 26 weeks, HFD, *ad libitum* fed, n = 6). (**H**) Lipidomic analysis of lysophosphatidylcholine (lysoPC) species in the liver (M, 26 weeks, *ad libitum* fed, n = 6). LysoPC (16:0) hydrolase activity assay using (**I**) microsomal liver fractions (M, 26 weeks, *ad libitum* fed, n = 6) and (**J**) purified recombinant Ces2a protein (n = 3). Data represent mean + SEM. Statistical significance was determined by A-G, I, J) Student's two-tailed *t* test or H) non-parametric Wilcoxon rank sum test in combination with the Benjamin-Hochburg procedure for multiple test correction. P values compare effect of genotype: ∗P < 0.05, ∗∗P < 0.01, ∗∗∗P < 0.001.

Although purified recombinant Ces2a protein has shown robust TAG and DAG hydrolytic activities *in vitro* [11], microsomal liver fraction of HFD-fed Ces2a-ko mice exhibited only a moderate and non-significant reduction of hydrolytic activities against TAGs and *sn*-1,3 DAGs (Figure 4A, B), but a significant reduction against *sn*-1,2 DAGs (Figure 4B). Hepatic mRNA expression of other hydrolases involved in intracellular lipid degradation such as *Hsl*, *Lal*, *Ces1d, Ces1g,* and *Ces2e* were similar between the genotypes except for an increase in *Atgl* and a decrease in *Ces2c* mRNA expression in Ces2a-ko liver (Figure S2B and C). Since carboxylesterases (e.g., Ces1d, Ces1g) have been implicated in VLDL secretion [19,20], we determined VLDL secretion rates in Ces2a-ko mice fed HFD which may interfere with hepatic lipid homeostasis (Fig. S2D). Plasma TAG levels following LPL inhibition were comparable between genotypes, indicating that augmented lipid deposition in Ces2a-ko liver does not originate from impaired VLDL secretion and/or changes in Ces1d/1g activities. Furthermore, expression of target genes involved in beta oxidation were either unchanged *(Pparα, Pgc1α, Aox1, Mcad)* or slightly increased (*Cpt1α, Lcad*) in the Ces2a-ko liver (Fig. S2E), suggesting rather a compensatory effect to counteract lipid accretion.

Besides reduced *sn*-1,2 DAG hydrolytic activity, hepatic lipid accumulation of HFD-fed Ces2a-ko mice was associated with increased mRNA expression of the fatty acid transporters *Cd36* and *Fabp4* (Figure 4C). In line with the increased expression of these two PPAR gamma (PPARg) target genes, hepatic *Ppar*g expression was elevated in response to Ces2a deficiency (Figure 4D). Notably, although mRNA expression of *Acc1* and *Fasn,* two key regulators of *de novo* lipogenesis, was slightly elevated (Figure 4D), protein expression of ACC, FAS as well as SCD1 remained unchanged (Figure 4E). In line with increased *Pparg* expression, mRNA expression of *Mgat1*, another PPARg target gene, was significantly increased together with elevated MGAT activity in the liver of HFD-fed Ces2a-ko mice (Figure 4D, F). Similarly, DGAT activity was also increased in Ces2a-ko microsomal liver fraction (Figure 4G) although *Dgat1* and *Dgat2* gene expression was comparable between the genotypes (Figure 4D). A recent study demonstrated a negative correlation of CES2 expression and intracellular lysophosphatidylcholine (lysoPC) and DAG levels [21,22] in cancer cell lines and another study discovered lysoPC (16:0) as a PPARg ligand [23]. Hence Ces2a deficiency may impact lysoPC levels as well. In line with this assumption, lipidomic analyses revealed significantly increased levels of lysoPC species (16:0, 16:1, 18:0, 18:1, and 20:4) in the liver of Ces2a-ko mice (Figure 4H), which was accompanied by reduced hepatic lysoPC hydrolytic activity in Ces2a-ko mice (Figure 4I). Furthermore, purified recombinant Ces2a protein exhibited distinct hydrolytic activity towards lysoPC (16:0), lysoPC (18:0), and lysoPC (18:1) (Figure 4J and Fig. S2F), indicating that Ces2a is also a potent lysoPC hydrolase. Of note, recombinant Ces2a showed no hydrolytic activity towards multiple phospholipid classes including PA, PC, PE, PS, PI, and PG (Fig. S2F). Thus, hepatic DAG accumulation in HFD-fed Ces2a-ko mice is caused by reduced *sn*-1,2 DAG hydrolytic activity along with increased lipid synthesis presumably involving PPARg activation via Ces2a-mediated lysoPC accumulation.

### 3.4 DAG accumulation in Ces2a-ko mice is associated with impaired hepatic insulin signaling

**Figure 5:**
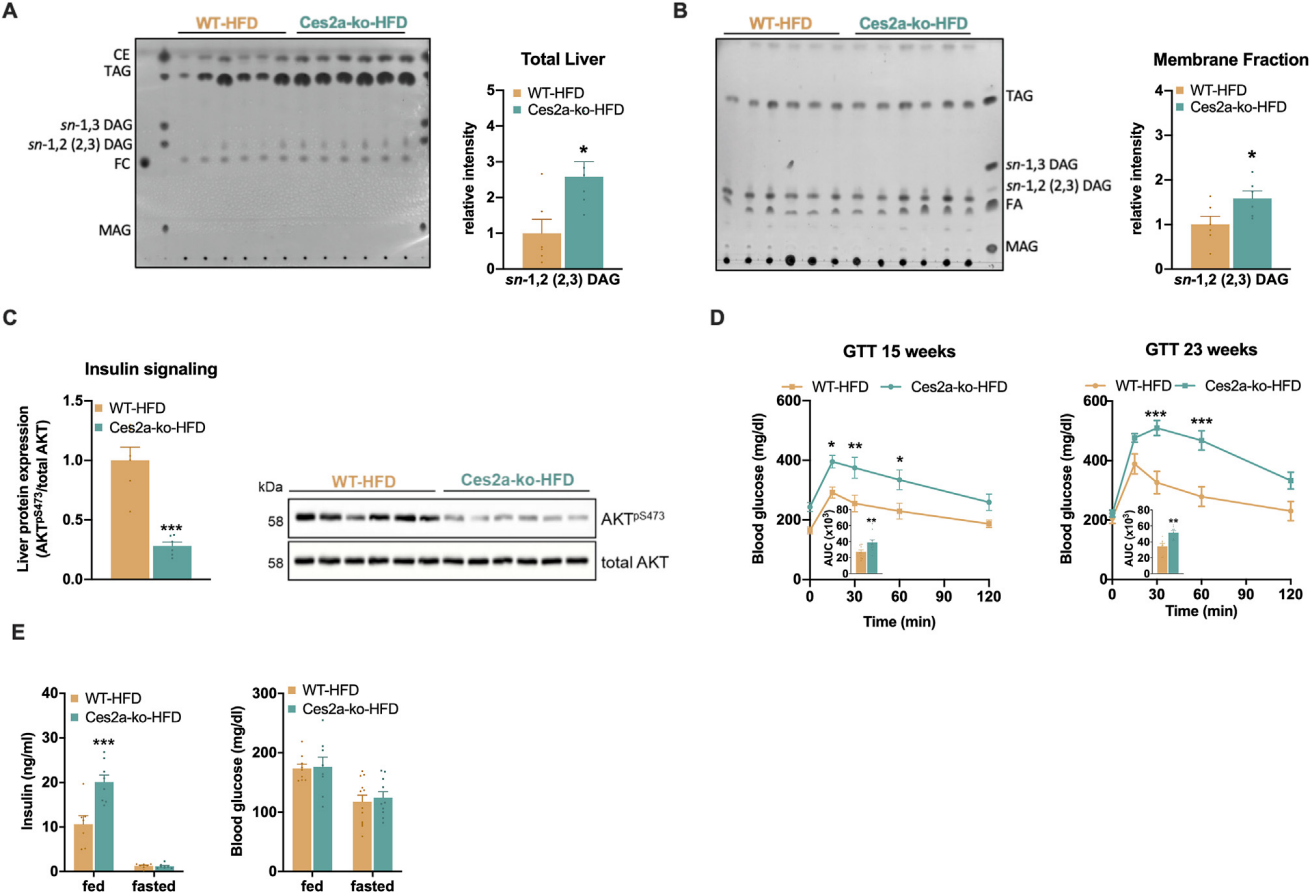
**Ces2a deficiency alters systemic and liver-specific glucose homeostasis and insulin action.** (**A**) Left: TLC of neutral lipids extracted from the liver. Right: Quantification of the *sn*-1,2 (2,3) diacylglycerol (DAG) levels (M, 26 weeks, HFD, *ad libitum* fed, n = 6). (**B**) Left: TLC of neutral lipids extracted from liver membrane fraction. Right: Quantification of the *sn*-1,2 (2,3) DAG levels (M, 26 weeks, HFD, *ad libitum* fed, n = 6). (**C**) Hepatic insulin signaling as a measure of AKT^pSer473^ levels. Left: Quantification of phosphorylation of AKT^pSer473^ relative to total AKT (M, 26 weeks, HFD, *ad libitum* fed, n = 6). Right: Representative immunoblots. (**D**) Glucose tolerance test (GTT) with 1.6 g glucose per kg body weight in 15- and 23-week old mice (M, HFD, 6 h fasted, n = 7–8). (**E**) Plasma insulin (left) and blood glucose levels (right; M, 26 weeks, HFD, *ad libitum* fed; 12 h fasted, n = 6–9). Data represent mean ± SEM. Statistical significance was determined by A, B, C) Student's two-tailed *t* test or D, E) two-way ANOVA followed by Bonferroni's *post hoc* analysis. P values compare effect of genotype: ∗P < 0.05, ∗∗P < 0.01, ∗∗∗P < 0.001.

Intracellular lipid accumulation is often linked to insulin resistance. Especially increased levels of *sn*-1,2 (2,3) DAGs are associated with impaired tissue-specific insulin sensitivity [24,25]. In agreement with reduced *sn*-1,2 DAG hydrolytic activity and increased MGAT activity, HFD-fed Ces2a-ko mice accumulated predominantly *sn*-1,2 (2,3) DAGs in liver tissue (Figure 5A). The increase in *sn*-1,2 (2,3) DAG deposition was also present in liver membrane fraction (Figure 5B). Higher *sn*-1,2 (2,3) DAG levels were accompanied by impaired hepatic insulin signaling shown by reduced phosphorylation of AKT S473 in the liver of Ces2a-ko mice (Figure 5C). In addition, phosphorylation of AKT S473 was or tended to be reduced in white and brown adipose tissue but was unchanged in skeletal muscle (Fig. S3A). In accordance, glucose clearance following a glucose bolus was declined in young and middle-aged HFD-fed Ces2a-ko mice (Figure 5D), indicating that Ces2a deficiency promotes glucose intolerance. Correspondingly, plasma insulin levels were elevated in HFD-fed Ces2a-ko mice in the fed state, whereas blood glucose levels were unchanged between the genotypes irrespective of feeding status further indicating reduced insulin sensitivity upon Ces2a deficiency (Figure 5E). Notably, despite massive lipid accumulation and impaired insulin signaling, expression and phosphorylation levels of genes and proteins characteristic for ER stress were comparable between HFD-fed Ces2a-ko and WT mice (Figure S3B and C). In line, ER morphology was similar between the genotypes (Fig. S3D), implicating that ER stress *per se* is not the main driver of hepatic insulin resistance in Ces2a-ko mice. Together, Ces2a deficiency impairs systemic glucose homeostasis at least partly by declined hepatic insulin sensitivity likely mediated by aberrant lipid signaling and *sn*-1,2 (2,3) DAG accumulation, respectively.

### 3.5 Inhibition of human CES2 activity in liver cells phenocopies the Ces2a-ko liver phenotype

**Figure 6:**
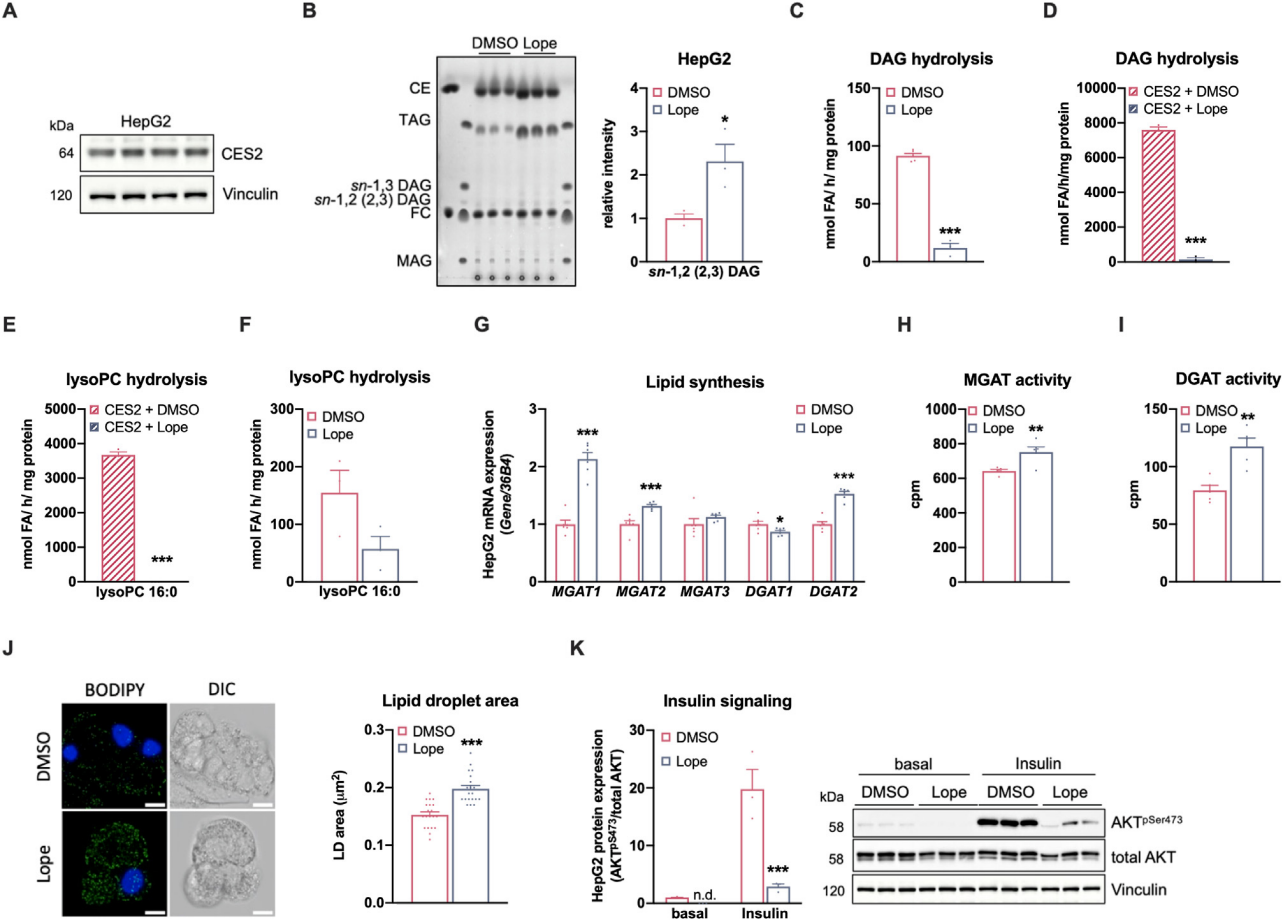
**Impact of CES2 inhibition on lipid metabolism and insulin signaling in HepG2 cells.** (**A**) Protein expression of CES2 in HepG2 cells (n = 4). (**B**) HepG2 cells were treated without (DMSO) or with 10 μM Loperamide (Lope) for 48 h. Left: TLC of neutral lipids extracted from HepG2 cells. Right: Quantification of intracellular *sn*-1,2 (2,3) diacylglycerol (DAG) levels (n = 3). (**C**) For DAG hydrolase activity assay, microsomal HepG2 cell lysates were treated acutely with 2 μM Loperamide (n = 3–5). (**D**) DAG hydrolase activity assay using purified recombinant CES2 protein in the absence or presence of 1 μM Loperamide (n = 3). (**E**) LysoPC (16:0) hydrolase activity assay using purified recombinant CES2 protein in the absence or presence of 1 μM Loperamide (n = 3). (**F**) For measuring lysoPC (16:0) hydrolase activity, microsomal preparations of HepG2 cells were treated acutely with 2 μM Loperamide (n = 5). HepG2 cells were treated without (DMSO) or with 10 μM Loperamide (Lope) for 48 h. (**G**) mRNA expression of genes involved in lipid synthesis relative to *36B4* reference gene by qPCR with DMSO-treated HepG2 cells arbitrarily set to 1 for each gene (n = 6). (**H**) MGAT and (**I**) DGAT activity assay using microsomal cell fractions (n = 5). (**J**) Left: Lipid droplet (LD) staining in HepG2 cells using BODIPY 493/503. scale bar: 10 μm. Right: Quantification of lipid droplet area. (**K**) Insulin signaling in HepG2 cells in the absence (basal) or presence of insulin (10 μg/ml). Left: Quantification of phosphorylation of AKT^pSer473^ relative to total AKT (n = 3). Right: Representative immunoblots. Data are representative of three independent experiments with a minimum of three biological replicates. Data represent mean + SEM. Statistical significance was determined by B-J) Student's two-tailed *t* test or K) two-way ANOVA followed by Bonferroni's *post hoc* analysis. P values compare effect of pharmacological treatment: ∗P < 0.05, ∗∗P < 0.01, ∗∗∗P < 0.001.

A previous study assigned Ces2c as the murine orthologue of CES2 [7]. However, due to similarities in substrate specificity and tissue-specific expression pattern of Ces2a and Ces2c, we have recently suggested that other Ces2 members could exert a CES2-related role in hepatocyte lipid metabolism in mice [11]. Given that Ces2a is an ortholog of human CES2, one would expect that CES2 inhibition provokes similar changes in hepatic lipid metabolism compared to Ces2a deficiency. To address this assumption, we examined the impact of Loperamide, a specific CES2 inhibitor [26], on lipid metabolism in HepG2 cells showing adequate endogenous CES2 protein expression (Figure 6A). Loperamide incubation significantly increased *sn*-1,2 (2,3) DAGs levels in HepG2 cells (Figure 6B). Particularly interesting, microsomal DAG hydrolytic activity was drastically reduced in Loperamide-treated cells (Figure 6C), corroborating an essential and limiting role of CES2 as microsomal DAG hydrolase in liver cells. In line and as previously shown [11], recombinant CES2 exhibited robust DAG hydrolase activity, which was blunted in the presence of Loperamide (Figure 6D). Furthermore, and similar to Ces2a, recombinant CES2 exhibited also a strong hydrolytic activity against lysoPC (Figure 6E and Fig. S2G), which again declined upon Loperamide treatment. Accordingly, CES2 inhibition reduced microsomal lysoPC hydrolytic activity in HepG2 cells (Figure 6F). Notably, recombinant CES2 showed no or negligible activity for PA, PC, and PE, but exhibited hydrolytic activity for PS, PI, and PG (Fig. S2G). In line with Ces2a deficiency, expression of genes involved in lipid synthesis (*MGAT1, MGAT2*, and *DGAT2*) and their corresponding MGAT and DGAT activities were increased in Loperamide-treated HepG2 cells compared to control cells (Figure 6G–I), resulting in increased lipid droplet area (Figure 6J). Finally, Loperamide-treated HepG2 cells exhibited impaired insulin signaling (Figure 6K) as present in the liver of Ces2a-ko mice. Together, inhibition of CES2 activity causes very similar changes in lipid and glucose metabolism of HepG2 cells as observed in the liver of Ces2a-ko mice.

### 3.6 Ectopic CES2 expression counteracts hepatic steatosis and improves glucose homeostasis in Ces2a-ko mice

**Figure 7:**
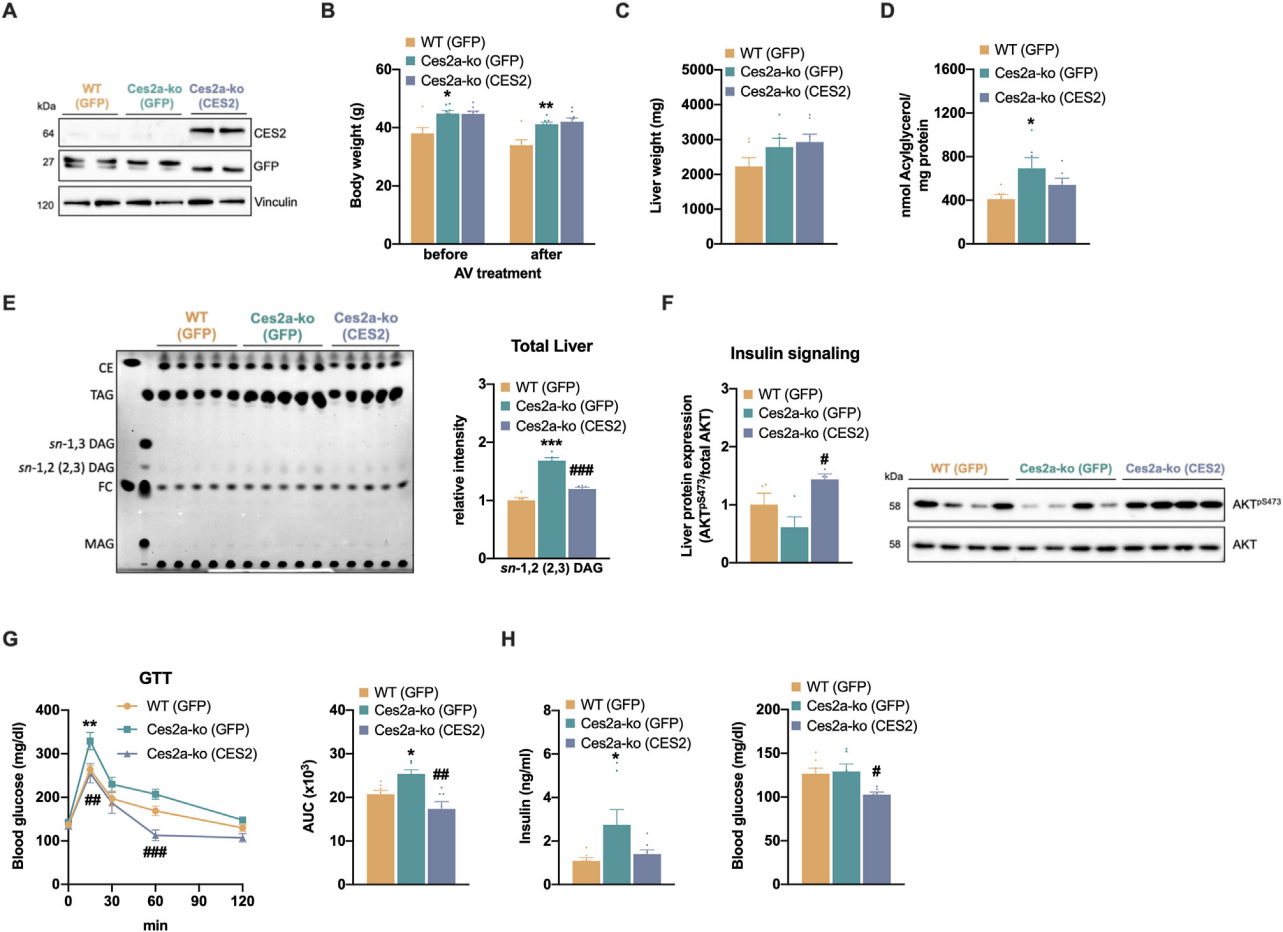
**Impact of ectopic CES2 expression on hepatic lipid and glucose metabolism in Ces2a-ko mice.** Mice were treated with adenovirus overexpressing GFP or CES2 for 8 days. (**A**) Protein expression of CES2 and GFP in the liver. (**B**) Body weight, (**C**) liver tissue weights and (**D**) hepatic total acylglycerol of adenoviral infected mice (M, 26 weeks, HFD, *ad libitum* fed, n = 6–7). (**E**) Left: TLC of neutral lipids extracted from the liver. Right: Quantification of *sn*-1,2 (2,3) DAG levels (M, 26 weeks, HFD, *ad libitum* fed, n = 6). (**F**) Hepatic insulin signaling as a measure of AKT^pSer473^ levels. Left: Quantification of phosphorylation of AKT^pSer473^ relative to total AKT (M, 26 weeks, HFD, *ad libitum* fed, n = 4). Right: Representative immunoblots. (**G**) Left: Glucose tolerance test (GTT) with 1.6 g glucose per kg body weight. Right: AUC of the GTT (M, 26 weeks, HFD, 6 h fasted, n = 6). (**H**) Plasma insulin (left) and blood glucose levels (right) (M, 26 weeks, HFD, *ad libitum* fed, n = 6–7). Data represent mean ± SEM. Statistical significance was determined by B, G) two-way ANOVA or C, D, E, F, H) one-way ANOVA followed by Tukey's *post hoc* analysis. P values compare effect of genotype (GFP vs GFP): ∗P < 0.05, ∗∗P < 0.01, ∗∗∗P < 0.001. P values compare effect of AV treatment (GFP vs CES2) in Ces2a-ko mice ^#^P < 0.05, ^##^P < 0.01, ^###^P < 0.001.

A recent study demonstrated reduced hepatic DAG levels and improved insulin signaling upon adenoviral overexpression of CES2 in HFD-fed WT mice [8]. These findings prompted us to inject CES2 recombinant adenovirus into the tail vein of Ces2a-ko mice to investigate the impact of ectopic CES2 expression on lipid and glucose homeostasis in Ces2a-ko mice (Ces2a-ko (CES2)). As controls, we injected recombinant GFP adenovirus into WT (WT (GFP)) and Ces2a-ko mice (Ces2a-ko (GFP)) (Figure 7A). AV treatment had no effect on body weight of WT and Ces2a-ko mice (Figure 7B). Liver weight was not different between the groups following adenovirus treatment, although the liver of WT (GFP) mice tended to be reduced compared to Ces2a-ko mice (Figure 7C). As expected, Ces2a-ko (GFP) mice had higher levels of hepatic acylglycerol compared to WT (GFP) mice. CES2 overexpression in Ces2a-ko mice reduced hepatic acylglycerol levels albeit values did not reach statistical significance (Figure 7D). Nevertheless, *sn*-1,2 DAG levels were markedly reduced in the liver of Ces2a-ko (CES2) mice compared to Ces2a-ko (GFP) mice and levels were comparable to WT (GFP) mice (Figure 7E). The reduction of *sn-*1,2 DAGs was accompanied by improved insulin signaling (Figure 7F), enhanced glucose clearance following a glucose bolus (Figure 7G) and lower plasma insulin and blood glucose levels in Ces2a-ko (CES2) mice compared to Ces2a-ko (GFP) mice (Figure 7H). Together, ectopic CES2 expression in Ces2a-ko mice reverses DAG accumulation and improves glucose clearance to WT level, suggesting that Ces2a exerts a closely related role in liver lipid metabolism compared to human CES2.

## 4 Discussion

The mechanisms causing hepatic steatosis and inflammation in obesity are incompletely understood. Recently, human CES2 and murine Ces2c have been discovered as metabolic lipases showing reduced mRNA expression in the liver of NASH patients and in obese mouse models exhibiting NAFLD [7,8,27,28]. Ces2a is the most abundantly expressed Ces2 member in the liver of mice and its expression levels are several-fold higher compared to Ces2c [9,11]. Moreover, hepatic Ces2a expression significantly declined in mice fed HFD or upon injection of CES2 recombinant adenovirus into WT mice [8,11], implicating that Ces2a may exert a CES2-related role in hepatic lipid metabolism. To investigate the role of Ces2a in lipid catabolism *in vivo*, we generated and characterized Ces2a-ko mice. Ces2a deficiency provokes obesity, glucose intolerance and severe hepatic steatosis when fed HFD accompanied by increased inflammatory gene expression and liver damage. Likewise, Ruby et al. [8] showed markedly reduced serine hydrolase activity of CES2 in the liver of obese individuals without NAFLD, implicating that reduced CES2 (and conceivably Ces2a) expression is an early event in NAFLD/NASH progression. Particularly interesting, the most prominent change in hepatic lipid composition, either in obese individuals, HFD-fed WT or in our Ces2a-ko mice is substantial DAG accumulation in the liver. To unravel the origin of DAG accumulation in obesity is a prerequisite to understand the metabolic changes provoking NAFLD/NASH development and insulin resistance in humans (and in Ces2a-ko mice).

Several Ces2 members and CES2 have been shown to act as TAG, DAG and MAG hydrolytic enzymes [7,8,11], and the conserved ER retention signal [29] suggests that these lipolytic enzymes are involved in lipid hydrolysis of LDs generated in the ER membrane and/or LDs located in the ER lumen [30]. Increased LD formation in the ER has been proposed to enhance ER stress in obesity, which in turn activates SREBP-1c processing and *de novo* lipogenesis [31] thereby enhancing TAG accumulation in a futile and deleterious cycle. Low hepatic Ces2c expression has been proposed to increase TAG levels in the ER as a consequence of impaired TAG hydrolysis ultimately activating SREBP-1c processing and *de novo* lipogenesis [7]. Contrasting Ces2c knockdown, expression of genes characteristic for ER stress and ER morphology were comparable among HFD-fed Ces2a-ko and WT mice, implicating that ER stress and likely SREBP-1c activation are not the main cause of severe hepatic steatosis and DAG accumulation in Ces2a deficiency. In accordance, protein levels of genes required for *de novo* lipogenesis including ACC, FAS, and SCD1 were comparable among Ces2a-ko and WT mice. In contrast, our study and the study by Ruby et al. [8] deliver several lines of evidence that Ces2a and CES2 play a critical role in liver microsomal DAG catabolism: i) Ces2a deficiency reduces microsomal DAG hydrolytic activity and significantly increases microsomal DAG levels in HFD-fed mice; ii) HFD-induced DAG accumulation in WT mice [8] and in Ces2a-ko mice could be reversed upon ectopic CES2 expression; iii) Pharmacological inhibition of CES2 enzymatic activity in HepG2 cells strongly reduced microsomal DAG hydrolytic activity paralleled by significantly increased *sn*-1,2 (2,3) DAG levels. In accordance, two recent studies linked low CES2 expression in cancer cell lines to DAG (and lysoPC) accumulation, which could be reversed upon CES2 overexpression, albeit these studies show a divergent impact of CES2 expression levels and the overall survival of cancer patients [21,22]. The stereochemical structure of DAGs determines their potential role in lipid synthesis and signaling processes. *sn-1,*2 DAG is the precursor of DGAT1-mediated glycerophospholipid synthesis at the ER [25,32] and solely *sn*-1,2 DAGs are able to activate various PKC isoforms [33]. Although controversially discussed [34], hepatic accumulation of *sn*-1,2 DAG has been suggested to trigger PKCε activation and subsequently the development of hepatic insulin resistance [24]. The significant accumulation of *sn*-1,2 (2,3) DAGs in the liver of Ces2a-ko mice likely induces PKC activation and thereby adversely interferes with insulin receptor substrate and AKT phosphorylation. In contrast to the robust increase in hepatic DAG species in Ces2a-ko mice, ceramide levels were similar to WT except for an increase in C20 and C24:1 ceramides, indicating a rather moderate if any impact of altered ceramide levels on hepatic insulin signaling. The role of ceramides in the development of NAFLD and insulin resistance is not as clear as compared to DAG. In rodents the reversal of hepatic steatosis is accompanied by reduced hepatic DAG levels and improved insulin action, whereas total hepatic ceramide levels were unchanged [35]. Our study suggests that altered lipid signaling, i.e. increased hepatic DAG (and lysoPC) levels adversely impact liver insulin signaling in Ces2a-ko mice. However, unchanged glucose levels (despite higher circulating insulin) and the moderate changes in adipose tissue insulin signaling suggest a more complex role of Ces2a deficiency in glucose homeostasis. Further clarification of the role of Ces2a deficiency in systemic glucose metabolism and insulin signaling requires euglycemic hyperinsulinemic clamp studies, which will be our focus in future studies.

Besides DAG accumulation, we found significantly increased lysoPC levels in the liver of Ces2a-ko mice. Interestingly, elevated lysoPC levels have been demonstrated in NASH patients and functionally linked to hepatocyte stress, lipo-apoptosis and insulin resistance [[36], [37], [38]]. Moreover, two recent studies associated low CES2 expression to lysoPC accumulation in human cancer cell lines [21,22]. However, in these studies, no hydrolytic activities were investigated, and the authors speculated that CES2 hydrolyzes PC and other phospholipids leading to lysoPC accumulation. Here, we demonstrate that recombinant Ces2a and CES2 exhibit significant hydrolytic activity towards lysoPC leading to reduced lysoPC hydrolytic activity in liver microsomal preparations of Ces2a-ko mice and Loperamide-treated HepG2 cells, suggesting that Ces2a and CES2 are involved in hepatic lysoPC catabolism. In contrast, we found no PC hydrolase activity for recombinant Ces2a and CES2, suggesting a specific role for these lipases in lysoPC catabolism. Particularly interesting, a recent study discovered lysoPC as an efficient PPARg agonist and administration of lysoPC in diabetic mice causes similar metabolic changes as observed after administration of rosiglitazone, an established PPARg agonist [39]. Although speculative, lysoPC accumulation in Ces2a deficiency may play a functional role in the induction of PPARg-target gene expression including genes involved in hepatic FA uptake (*Cd36*) and DAG production (*Mgat1*). Nevertheless, increased adiposity in Ces2a-ko mice might also interfere with hepatic PPARg activation via increased adipose tissue FA flux to the liver. Hepatic DAG and TAG can be synthesized via acylation of glycerol-3-phosphate or alternatively via the MGAT pathway. MGAT and DGAT enzymatic activities were significantly increased in liver microsomes prepared from Ces2a-ko mice, suggesting that DAG and TAG accumulation in Ces2a-ko mice involves the MGAT/DGAT pathway besides impaired DAG catabolism. In line with this assumption, hepatic overexpression of DGAT2 markedly increased DAG levels in the liver of the transgenic mice [24] and knockdown of MGAT1 expression protected mice from HFD and PPARg-induced hepatic steatosis, respectively [40]. Moreover, reduced expression of MGAT encoding genes has been demonstrated in NAFLD patients [41], implicating the relevance of this pathway in humans. In accordance, pharmacological inhibition of CES2 in human HepG2 cells largely phenocopies the alterations present in the liver of Ces2a-ko mice encompassing *sn*-1,2 (2,3) DAG and TAG accumulation together with increased MGAT and DGAT activity. The drastic decline in microsomal DAG hydrolytic activity upon CES2 inhibition in human HepG2 cells suggests that CES2 is likely the rate-limiting microsomal DAG hydrolase in humans.

Finally, we showed that HFD-induced hepatic DAG accumulation and glucose intolerance in Ces2a-ko mice can be reversed upon ectopic CES2 expression, suggesting that Ces2a is a functional ortholog of CES2 and that Ces2a-ko mice are a validated mouse model for preclinical studies. An interesting feature of Ces2a deficiency is adiposity even in chow-fed mice and the absence of hepatic fat accumulation. Besides the liver, Ces2a is also robustly expressed in the small intestine [9,11] and it is conceivable that the systemic lack of Ces2a provokes the development of obesity via aberrant intestinal lipid metabolism. In line with such a scenario, we have recently shown that intestine-specific overexpression of Ces2c, a homologue of Ces2a, counteracts HFD-induced obesity via increased lipid hydrolysis in the small intestine and changes in chylomicron size and clearance [27]. The similar lipid hydrolytic activities of Ces2c compared to Ces2a and the partially overlapping alterations in hepatic lipid metabolism in Ces2c and Ces2a knockdown/deficiency suggest that these lipases could act in concert as demonstrated for ATGL and HSL in TAG and DAG hydrolysis [42], respectively.

### 4.1 Conclusion

Our study elucidates Ces2a and CES2 as microsomal DAG and lysoPC hydrolases *in vivo* and suggests that aberrant hepatic lipid signaling due to decreased hepatic CES2/Ces2a expression is an important driver in the development of obesity-induced NAFLD and insulin resistance in humans and mice.

## Authors contributions

Gabriel Chalhoub: Investigation, Methodology, Formal analysis, Visualization, Software, Writing - Original Draft. Alina Jamnik: Investigation, Methodology, Visualization, Formal analysis, Writing - Original Draft. Laura Pajed: Investigation, Methodology, Visualization, Formal analysis. Stephanie Kolleritsch: Investigation, Methodology. Victoria Hois: Investigation, Methodology. Antonia Bagaric: Investigation. Dominik Prem: Investigation, Visualization. Anna Tilp: Investigation. Dagmar Kolb: Investigation, Visualization. Heimo Wolinski: Investigation, Visualization, Formal analysis. Ulrike Taschler: Investigation, Methodology. Thomas Züllig: Investigation, Formal analysis, Software. Gerald N. Rechberger: Investigation, Formal analysis. Claudia Fuchs: Investigation, Visualization, Methodology. Michael Trauner: Methodology, Conceptualization. Gabriele Schoiswohl: Supervision, Project administration, Conceptualization, Methodology, Writing - Original Draft, Review & Editing, Visualization. Guenter Haemmerle: Supervision, Project administration, Conceptualization, Methodology, Writing - Original Draft, Review & Editing.

## Funding information

This work was supported by the following: Austrian Science Fund (FWF): SFB F73 (GH, MT, GS, UT), P28882-B21 (GS), P31638-B34 (UT), DK MCD Metabolic and cardiovascular diseases W 1226 (GH); DOC fellowship 25049 (LP) funded by the Austrian Academy of Science (OEAW).

## Acknowledgement

We gratefully acknowledge the following contributions: We thank Kathrin A. Zierler, Birgit Juritsch, and Sharon Fiorin for animal care and genotyping as well as Dominique Pernitsch and Valentina Telser for technical assistance. We also acknowledge the support of the field of excellence BioHealth.

## Conflict of interest

None declared

## Data Availability

Data will be made available on request.
