# Supplementary material for: Carboxylesterase 2a deletion provokes hepatic steatosis and insulin resistance in mice involving impaired diacylglycerol and lysophosphatidylcholine catabolism
Source: Mol Metab. 2023 Apr 12;72:101725. doi: 10.1016/j.molmet.2023.101725 (PMC10148186; doi:10.1016/j.molmet.2023.101725)
Supplement: Multimedia component 2 [file mmc2.docx]

**SUPPLEMENTARY INFORMATION**

**Carboxylesterase 2a deletion provokes hepatic steatosis and insulin resistance in mice involving impaired diacylglycerol and lysophosphatidylcholine catabolism**

Gabriel Chalhoub^1*^, Alina Jamnik^1*^, Laura Pajed^1^, Stephanie Kolleritsch^1^, Victoria Hois^2^, Antonia Bagaric^3^, Dominik Prem^1^, Anna Tilp^1^, Dagmar Kolb^4^, Heimo Wolinski^1^, Ulrike Taschler^1^, Thomas Züllig^1^, Gerald N. Rechberger^1^, Claudia Fuchs^5^, Michael Trauner^5^, Gabriele Schoiswohl^3#^, Guenter Haemmerle^1#^

^1^Institute of Molecular Biosciences, University of Graz, Graz, Austria

^2^Division of Endocrinology and Diabetology, Medical University of Graz, Austria

^3^Department of Pharmacology and Toxicology, University of Graz, Graz, Austria

^4^Core Facility Ultrastructure Analysis, Medical University of Graz, Graz, Austria

^5^Hans Popper Laboratory of Molecular Hepatology, Division of Gastroenterology and Hepatology, Department of Internal Medicine III, Medical University of Vienna, Vienna, Austria

^*^ contributed equally, ^#^ corresponding authors

**SUPPLEMENTARY TABLES**

**Table S1. Primary antibodies**

| **Protein** | **Protein name** | **Company** | **Dilution used** |
| --- | --- | --- | --- |
| ACC | Acetyl-CoA carboxylase 1 | Cell Signaling, 3676 | 1:2,000 in 5% BSA |
| AKT | Protein kinase B | Cell Signaling, C67E7 | 1:1,000 in 5% BSA |
| AKT pSer473 | Phospho-Protein kinase B | Cell Signaling, 9271S | 1:1,000 in 5% BSA |
| ATGL | Adipose triglyceride lipase | Cell Signaling, 2138 | 1:1,000 in 5% milk |
| BIP | Immunoglobulin heavy chain-binding protein | Cell Signaling, 3177 | 1:1,000 in 5% BSA |
| CES2 | Carboxylesterase 2 | Thermo Scientific, PA-534842 | 1:2,000 in 5% milk |
| eIF2a | Eukaryotic initiation factor-2α | Cell Signaling, 9722 | 1:1,000 in 5% BSA |
| eIF2a pSer51 | Phospho-Eukaryotic initiation factor-2α | Cell Signaling, 9721S | 1:1,000 in 5% BSA |
| FAS | Fatty acid synthase | Cell Signaling, 3180 | 1:3,000 in 5% BSA |
| GFP | Green-fluorescent protein | Abcam, 6556 | 1:4,000 in 5% milk |
| HSL | Hormone sensitive lipase | Cell Signaling, 4107 | 1:1,000 in 5% BSA |
| IRE1a | Inositol-Requiring Enzyme 1 α | Cell Signaling, 3294 | 1:1,000 in 5% BSA |
| SCD1 | Stearoyl-CoA desaturase 1 | Cell Signaling, 2438 | 1:2,000 in 5% BSA |
| Vinculin | Vinculin | Sigma Aldrich, V9131 | 1:5,000 in 5% BSA |

**Table S2. Primer sequences used for quantitative gene expression analysis**

| **Gene** | **Gene name** | **Primer sequence** |
| --- | --- | --- |
| *Acc1* | Acetyl-CoA carboxylase 1 | fw: GCCAGCTCTGGAGGTGTATG  rv: TCCTTAAGCTGGCGGTGTT |
| *Aox1* | Acyl-CoA oxidase 1 | fw: AGATTGGTAGAAATTGCTGCAAAA  rv: ACGCCACTTCCTTGCTCTTC |
| *Atf4* | Activating transcription factor 4 | fw: GGACAGATTGGATGTTGGAGAAAATG  rv: GGAGATGGCCAATTGGGTTCAC |
| *Atf6* | Activating transcription factor 6 | fw: TTATCAGCATACAGCCTGCG  rv: CTTGGGACTTTGAGCCTCTG |
| *Atgl* | Adipose triglyceride lipase | fw: GAG ACC AAG TGG AAC ATC  rv: GTA GAT GTG AGT GGC GTT |
| *α-Sma* | Alpha-smooth muscle actin | fw: GGCTCTGGGCTCTGTAAGG  rv: CTCTTGCTCTGGGCTTCATC |
| *Cd11c* | Cluster of differentiation 11c | fw: CAGTGACCCCGATCACTCTT  rv: CACCACCAGGGTCTTCAAGT |
| *Cd36* | Cluster of differentiation 36 | fw: GAACCTATTGAAGGCTTACATCC  rv: CCCAGTCACTTGTGTTTTGAAC |
| *Ces1d* | Carboxylesterase 1d | fw: ATATGGCTTTCTCTTGCTGCG  rv: CCCAGGACTTTGCCTTTAACAGT |
| *Ces1g* | Carboxylesterase 1g | fw: CGAGTCAGCAGGAGGTGAAAGT  rv: TTGAAAATGACACTACTCTGAGCG |
| *Ces2a* | Carboxylesterase 2a | fw: CTCACAGCCGGCCATGT  rv: AGATTCATTTCCTTCGCATCCT |
| *Ces2c* | Carboxylesterase 2c | fw: GCTGAATGCTGGGTTCTTCG  rv: GCTGCCTTGGATCTGTCCTGT |
| *Ces2e* | Carboxylesterase 2e | fw: CTTGTCTTTGGCTACCAGTTCG  rv: TTGCTCCTCTTCCTCAGTGTAAGG |
| *Col1a1* | Collagen Type 1 alpha 1 | fw: CCGGCTCCTGCTCCTCCTA  rv: CCATTGTGTATGCAGCTGACTTC |
| *Col1a2* | Collagen Type 1 alpha 2 | fw: AAGGGTGCTACTGGACTCCC  rv: TTGTTACCGGATTCTCCTTTGG |
| *Cpt1α* | Carnitine o-palmitoyltransferase 1 | fw: CACCAACGGGCTCATCTTCTA  rv: CAAAATGACCTAGCCTTCTATCGAA |
| *Dgat1* | Diacylglycerol o-acyltransferase 1 | fw: GTGCACAAGTGGTGCATCAG  rv: CAGTGGGATCTGAGCCATCA |
| *Dgat2* | Diacylglycerol o-acyltransferase 2 | fw: TTCCTGGCATAAGGCCCTATT  rv: AGTCTATGGTGTCTCGGTTGAC |
| *Elovl6* | Elongation of very long chain fatty acids protein 6 | fw: TCAGCAAAGCACCCGAAC  rv: AGCGACCATGTCTTTGTAGGAG |
| *Fabp4* | Fatty acid binding protein 4 | fw: GAACCTGAAGCTTGTCTTCG  rv: ACCAGCTTGTCACCATCTCG |
| *Fasn* | Fatty acid synthase | fw: TGC TCC CAG CTG CAG GC  rv: GCC CGG TAG CTC TGG GTG TA |
| *F4/80* | Cell surface glycoprotein F4/80 | fw: GGATGTACAGATGGGGGATG  rv: CATAAGCTGGGCAAGTGGTA |
| *Hsl* | Hormone-sensitive lipase | fw: GCTGGGCTGTCAAGCACTGT  rv: GTAACTGGGTAGGCTGCCAT |
| *Il1ß* | Interleukin-1 beta | fw: CACAGCAGCACATCAACAAG  rv: GTGCTCATGTCCTCATCCTG |
| *Il6* | Interleukin-6 | fw: GAGGATACCACTCCCAACAGACC  rv: AAGTGCATCATCGTTGTTCATACA |
| *Lal* | Lysosomal acid lipase | fw: GGCGGAAGAACCATTTTGG  rv: GCAAGCCGTGCTGAAGATACA |
| *Lcad* | Long-chain specific acyl-CoA dehydrogenase | fw: TTTCCGGGAGAGTGTAAGGA  rv: ACTTCTCCAGCTTTCTCCCA |
| *Lpl* | Lipoprotein lipase | fw: GGGAGTTTGGCTCCAGAGTTT  rv: TGTGTCTTCAGGGGTCCTTAG |
| *Lxra* | Liver X receptor alpha | fw: CTCAATGCCTGATGTTTCTCCT  rv: TCCAACCCTATCCCTAAAGCAA |
| *Mcad* | Medium-chain specific acyl-CoA dehydrogenase | fw: GCAACTGCCCGCAAGTTT  rv: TACTCCCCGCTTTTGTCATATTC |
| *Mgat1* | Monoacylglycerol acyltransferase 1 | fw: GAGTAACGGGCCGGTTTC A  rv: AGACATTGCCACCTCCATCCT |
| *Pgc1α* | Peroxisome proliferator-activated receptor gamma coactivator 1-alpha | fw: CCCTGCCATTGTTAAGACC  rv: TGCTGCTGTTCCTGTTTTC |
| *Pparα* | Peroxisome proliferator-activated receptor alpha | fw: GTACCACTACGGAGTTCACGCAT  rv: CGCCGAAAGAAGCCCTTAC |
| *Pparg* | Peroxisome proliferator-activated receptor gamma | fw: AACAAGACTACCCTTTACTGAAATTACCA  rv: CACAGAGCTGATTCCGAAGTTG |
| *Scd1* | Stearoyl-CoA desaturase-1 | fw: TGGGTTGGCTGCTTGTG  rv: GCGTGGGCAGGATGAAG |
| *Srebp1c* | Sterol regulatory element-binding protein 1 | fw: GTTACTCGAGCCTGCCTTCAGG  rv: CAAGCTTTGGACCTGGGTGTG |
| *Tnfα* | Tumor necrosis factor alpha | fw: GACCCTCACACTCAGATCATCTTCT  rv: CCTCCACTTGGTGGTTTGCT |
| *Xbp1* | X-box binding protein 1 (spliced) | fw: GGTCTGCTGAGTCCGCAGCAGG  rv: AGGCTTGGTGTATACATGG |
| *Xbp1* | X-box binding protein 1 (total) | fw: TTGTCACCTCCCCAGAACATC  rv: TCCAGAATGCCCAAAAGGAT |
| *36B4* | Acidic ribosomal phosphoprotein P0 | fw: GCTTCATTGTGGGAGCAGACA  rv: CATGGTGTTCTTGCCCATCAG |
| *DGAT1* | Diacylglycerol o-acyltransferase 1 | fw: GCTTCAGCAACTACCGTGGCAT  rv: CCTTCAGGAACAGAGAAACCACC |
| *DGAT2* | Diacylglycerol o-acyltransferase 2 | fw: CTACAGGTCATCTCAGTGCT  rv: GAAGTAGAGCACAGCGATGA |
| *MGAT1* | Monoacylglycerol acyltransferase 1 | fw: AAAGTGTGTCCTACATGGTAAGC  rv: TGATCCTTCAGGGTTGTCAGTT |
| *MGAT2* | Monoacylglycerol acyltransferase 2 | fw: CCTTCGGGGAGAATGACCTA  rv: GAGGGAGATGCCCATGATCTT |
| *MGAT3* | Monoacylglycerol acyltransferase 3 | fw: ATGGGAGTTGCCACAACCC  rv: CAGAGTGACGTGAAGAGGAGG |
| *PPARg* | Peroxisome proliferator-activated receptor gamma | fw: TGGAATTAGATGACAGCGACTTGG  rv: CTGGAGCAGCTTGGCAAACA |

**Table S3. Plasma parameters of WT and Ces2a-ko mice fed chow diet**

Plasma parameters of WT and Ces2a-ko mice fed chow diet (CD; M, 28 weeks, fasted 12 h, 2 h refed n=5-8). Data represent mean ± SEM. Statistical significance was determined by Student’s two-tailed t test. P values compare effect of genotype: P < 0.05: *.

|  | WT-CD | | Ces2a-ko-CD | | WT-CD | Ces2a-ko-CD | |
| --- | --- | --- | --- | --- | --- | --- | --- |
| Parameter | | **fasted** | | | **refed** | | |
| Fatty acid (mM) | | 1.19 ± 0.07 | | 1.12 ± 0.08 | 0.48 ± 0.02 | | 0.55 ± 0.04 |
| Glycerol (mM) | | 0.49 ± 0.03 | | 0.45 ± 0.07 | 0.31 ± 0.03 | | 0.38 ± 0.02 |
| Triacylglycerol (mM) | | 0.78 ± 0.04 | | 0.81 ± 0.05 | 1.02 ± 0.11 | | 1.22 ± 0.09 |
| Total cholesterol (mM) | | 2.01 ± 0.09 | | 2.33 ± 0.04* | 1.91 ± 0.09 | | 2.29 ± 0.10* |

**SUPPLEMENTARY FIGURE LEGENDS**

**Figure S1. Impact of Ces2a deficiency on systemic energy homeostasis.** (**A**) Tissue-specific *Ces2a* mRNA expression relative to *36B4* reference gene by qPCR with *Ces2a* expression in WT liver arbitrarily set to 1. *Ces2a* mRNA expression in white and brown adipose tissue, kidney, pancreas, spleen, cardiac and skeletal muscle was not detectable in WT mice (M, 18 weeks, chow, *ad libitum* fed, n=4-6). (**B**) X-Gal staining of various tissues obtained from Ces2a-ko mice (M, 18 weeks, chow, *ad libitum* fed). (**C-E**) Metabolic phenotyping of mice using a laboratory animal monitoring system. (**C**) Energy expenditure (EE) per day calculated by the formula: heat x 0,859 845 227 858 99 kcal/h (1 Watt), (**D**) respiratory exchange ratio (RER), and (**E**) locomotor activity in mice fed chow (left) or HFD (right; M, 10 weeks, *ad libitum* fed, n=5-7). Data represent mean + SEM. Statistical significance was determined by A) Student’s two-tailed *t* test or C) one-way ANOVA followed by Tukey’s *post hoc* analysis or D, E) two-way ANOVA followed by Bonferroni’s *post hoc* analysis. P values compare effect of genotype: ***P < 0.001.

**Figure S2. Ces2a deficiency interferes with hepatic lipid metabolism.** (**A**) Lipidomic analysis of total ceramide levels (left) and ceramide species (right) in the liver (M, 26 weeks, HFD, *ad libitum* fed, n=6). Hepatic mRNA expression of (**B**) lipases and (**C**) carboxylesterases relative to *36B4* reference gene by qPCR with WT mice arbitrarily set to 1 for each gene (M, 26 weeks, HFD, *ad libitum* fed, n= 7-8). (**D**) VLDL secretion determined by plasma TAG levels followed an ip injection of 1 g Poloxamer 407 per kg body weight (M, 26 weeks, HFD, 12h fasted, n= 8-9). (**E**) Hepatic mRNA expression of genes involved in beta oxidation relative to *36B4* reference gene by qPCR with WT mice arbitrarily set to 1 for each gene (M, 26 weeks, HFD, *ad libitum* fed, n= 8-9). Phospholipid (PA, phosphatidic acid; PC, phosphatidylcholine; PE, phosphatidylethanolamine; PS, phosphatidylserine; PI, phosphatidylinositol; PG, phosphatidylglycerol) and lysoPC (18:0, 18:1) hydrolase activity assays using (**F**) purified recombinant Ces2a and (**G**) CES2 protein (n=3). Data represent mean ± SEM. Statistical significance was determined by B, C, E) Student’s two-tailed *t* test or A) non-parametric Wilcoxon rank sum test in combination with the Benjamin-Hochburg procedure for multiple test correction or D) two-way ANOVA followed by Bonferroni’s *post hoc* analysis. P values compare effect of genotype: *P < 0.05, **P < 0.01. n.d.=not detectable.

**Figure S3. Ces2a deficiency does not augment hepatic ER stress. (A) I**nsulin signaling in adipose tissue (brown, BAT, white, WAT [perigonadal],) and skeletal muscle (SM [quadriceps]) determined by phosphorylation of AKT^pSer473^. Left: Quantification of phosphorylation of AKT^pSer473^ relative to total AKT (M, 26 weeks, HFD, *ad libitum* fed, n=6). Right: Representative immunoblots. **(B**) Hepatic expression of proteins involved in ER stress. Left: Quantification of BIP and IRE1a relative to Vinculin and phosphorylation of eIF2a relative to total elF2a. Right: Representative immunoblots (M, 26 weeks, HFD, *ad libitum* fed, n=6). (**C**) Hepatic mRNA expression of genes involved in ER stress relative to 36B4 reference gene by qPCR with WT mice arbitrarily set to 1 for each gene (M, 26 weeks, HFD, *ad libitum* fed, n=6-9). (**D**) Representative electron micrograph of liver sections. Hepatocytes show lipid droplets (LD), rough endoplasmic reticulum (ER), mitochondria (M), and nucleus (N; scale bar: 2 μm). Data represent mean + SEM. Statistical significance was determined by Student’s two-tailed *t* test. P values compare effect of genotype: *P < 0.05.
